# Normobaric Hyperoxia for Treatment of Pneumocephalus after Posterior Fossa Surgery in the Semisitting Position: A Prospective Randomized Controlled Trial

**DOI:** 10.1371/journal.pone.0125710

**Published:** 2015-05-20

**Authors:** Bujung Hong, Frank Biertz, Peter Raab, Dirk Scheinichen, Philipp Ertl, Anika Grosshennig, Makoto Nakamura, Elvis J. Hermann, Josef M. Lang, Heinrich Lanfermann, Joachim K. Krauss

**Affiliations:** 1 Department of Neurosurgery, Hannover Medical School, Hannover, Germany; 2 Institute for Biostatistics, Hannover Medical School, Hannover, Germany; 3 Institute of Diagnostic and Interventional Neuroradiology, Hannover Medical School, Hannover, Germany; 4 Department of Anaesthesiology, Hannover Medical School, Hannover, Germany; The George Washington University, UNITED STATES

## Abstract

**Background:**

Supratentorial pneumocephalus after posterior fossa surgery in the semisitting position may lead to decreased alertness and other symptoms. We here aimed to prove the efficacy of normobaric hyperoxia on the absorption of postoperative pneumocephalus according to a standardized treatment protocol.

**Methods and Findings:**

We enrolled 44 patients with postoperative supratentorial pneumocephalus (> 30 ml) after posterior fossa surgery in a semisitting position. After randomisation procedure, patients received either normobaric hyperoxia at FiO_2_ 100% over an endotracheal tube for 3 hours (treatment arm) or room air (control arm). Routine cranial CT scans were performed immediately (CT1) and 24 hours (CT2) after completion of surgery and were rated without knowledge of the therapy arm. Two co-primary endpoints were assessed: (i) mean change of pneumocephalus volume, and (ii) air resorption rate in 24 hours. Secondary endpoints were subjective alertness (Stanford Sleepiness Scale) postoperatively and attention (Stroop test), which were evaluated preoperatively and 24 hours after surgery. The mean change in pneumocephalus volume was higher in patients in the treatment arm as compared to patients in the control arm (p = 0.001). The air resorption rate was higher in patients in the treatment arm as compared to patients in the control arm (p = 0.0015). Differences were more pronounced in patients aged 52 years and older. No difference between patients in treatment arm and control arm was observed for the Stroop test. The distribution of scores in the Stanford Sleepiness Scale differed in the treatment arm as compared to the control arm, and there was a difference in mean values (p = 0.015).

**Conclusions:**

Administration of normobaric hyperoxia at FiO_2_ 100% via an endotracheal tube for 3 hours is safe and efficacious in the treatment of pneumocephalus after posterior fossa surgery in the semisitting position. Largest benefit was found in elderly patients and particularly in older men.

**Trial Registration:**

German Clinical Trials Register DRKS00006273

## Introduction

Surgical interventions in the semisitting position are considered advantageous for certain posterior fossa tumors by reducing bleeding in the operation field, reducing brain swelling and venous congestion, allowing for a clean surgical site, and facilitating the surgical approach. There are, however, some potentially severe complications associated with surgery in the semisitting position such as venous air embolism. In addition, pneumocephalus is a common problem.

Air depots are thought to occur in up to 100% of patients after surgery in the semisitting position [1–9]. The development of pneumocephalus is caused by the”inverted pop bottle” phenomenon, that is air bubbles move to the top of the container (cranium) as cerebrospinal fluid (CSF) pours out [10]. Furthermore, administration of dehydrating agents (e.g. mannitol, furosemid), use of nitrous oxide (N_2_O), hyperventilation, removal of a space-occupying mass, cerebral hemorrhage-associated vasoconstriction, drainage of CSF, and the gravitational effect contribute to the development of pneumocephalus [9, 11, 12].

The frontal subdural space is the most common location of pneumocephalus [13]. Small amounts of intracranial air are reabsorbed completely within days or weeks, and in most instances, no clinical signs or symptoms are noted [4, 5]. Accumulation of large amounts of intracranial air can result in a neurosurgical emergency, particularly if the pressure of air increases (so-called tension pneumocephalus), which may result in brain compression with potentially life-threatening brainstem herniation.

Thus far, there are only few uncontrolled studies on measures to reduce pneumocephalus. Normobaric oxygen has been suggested to be effective to treat pneumocephalus, since oxygen is able to replace nitrogen by increasing the diffusion gradient for nitrogen between the air collection and the surrounding tissue [14].

Since there is no study yielding higher class evidence on the application of oxygen for treatment of pneumocephalus, we decided to perform a prospective randomized study. We hypothesized that normobaric hyperoxia at fraction of inspired oxygen (FiO_2_) of 100% might accelerate the resorption of postoperative supratentorial pneumocephalus after posterior fossa surgery in the semisitting position. Moreover, we also hypothesized, that this measure might accelerate the patient’s ability to regain attention and recovery from sleepiness after surgery. To achieve these goals, we randomized patients for administration of 100% oxygen (O_2_) over an endotracheal tube (ETT) for three hours after detection of postoperative pneumocephalus on cranial computer tomography (CT) scan.

## Methods

The protocol for this trial and supporting CONSORT checklist are available as supporting information; see S1 CONSORT Checklist and S2 Protocol.

### Study design and participants

A prospective, randomized, observer-blind, controlled clinical trial was planned to involve 44 adult patients with pneumocephalus after posterior fossa surgery in the semisitting position between August 1, 2010 and April 30, 2012. The study was approved by the ethics committee of Hannover Medical School on February 1, 2010. Prior to surgery, written informed consent for the study was obtained from all patients. This study was initiated in the interest of improvement of postoperative care for inpatients in our own department only by assessing treatment possibility of normobaric hyperoxia, therefore the registration in the German Clinical Trials Register was conducted after enrolment of patients started. The authors confirm that all ongoing and related trials for this intervention are registered.

Primary inclusion criteria were the need for posterior fossa surgery in the semi-sitting position (as performed routinely in the authors’ institution) and age above 18.

On admission, all patients underwent routine chest X-rays, pulmonary function test, and transthoracic echocardiography (TTE) to exclude persistent foramen ovale.

Patients with a history of cardiac disease or cardiac surgery, pulmonary disease, chronic obstructive pulmonary disease (COPD), chronic cough or dyspnoe, abnormal chest X-ray, or pulmonary surgery were not considered for this study.

All patients who were considered possible candidates for the study were informed that they would only be finally included in the study if a routine postoperative CT scan would show pneumocephalus defined as intracranial air collection of 30 ml or more. Patients who had a CSF drain placed or who needed prolonged postoperative ventilation were excluded. Also pregnant or breastfeeding women were excluded.

### Randomization and masking

To ensure admission before treatment allocation, a telephone randomization has been set up with the Institute of Biostatistics. Specifically, (i) baseline data (patient number, initials, gender, age) were reported via telephone and all inclusion and exclusion criteria were confirmed, (ii) patients were allocated to treatment groups according to a static list that was generated using random permuted blocks with variable length held confidential at the Institute of Biostatistics, (iii) each telephone randomisation was confirmed via fax by the study physician.

### Procedures

Standard anaesthetic procedures were performed in all patients. Oral midazolam was given for premedication. After preoxygenation for at least 2 minutes, anaesthesia was induced with propofol (2.5 mg/kg) and sufentanil (0.3 μg/kg), and a neuromuscular block was obtained with atracurium (0.5 mg/kg). After endotracheal intubation, mechanical ventilation was started with a tidal volume of 8 ml/kg body weight and the respiratory rate was set on 12 per minute.

A multi-orifice single-lumen central venous catheter was placed into the left atrium to allow air retrieval in case of venous air embolism. Routine intraoperative monitoring included 5 lead electrocardiography, capnography, pulse oxymetry, recording of central venous pressure (CVP), invasive and non-invasive arterial blood pressure, body temperature, and urine output. A precordial TTE was placed to recognize air embolism. A probatory test was performed by injecting 5 ml 0.9% saline through the central venous catheter.

After induction of anaesthesia, patients were preloaded with 10 ml/kg of a colloid solution prior to positioning. Throughout surgery adequate oxygenation and a partial arterial carbon dioxide pressure (paCO_2_) level between 30 and 35 mmHg were secured. All patients received positive end expiration pressure (PEEP) at 5 cm H_2_O. A heating blanket was used to keep body temperature at 37°C. No N_2_O or mannitol was administered during surgery. Arterial blood gas (ABG) was analyzed regularly every 2 hours and after closure of craniotomy. Anaesthesia was maintained by continuous infusion of remifentanil (30μg/kg per hour) and propofol (10mg/kg per hour).

Patients were positioned in the semisitting position with the head flexed and rotated 30° toward the affected side. The legs were elevated at heart level, and the knees were slightly flexed. The head was fixed in a standard three-pin Mayfield clamp.

Standard intraoperative multimodal monitoring included somatosensory evoked potentials (SSEP). electromyography (EMG), and brainstem auditory evoked potential (BAEP) dependent on the localization of the lesion. Tumors were removed by standard microsurgical procedures.

A postoperative cranial CT scan was performed immediately after completion of surgery to detect postsurgical complications as well as to determine the presence and the volume of intracranial air. Anaesthesia was maintained as intraoperatively during transport from the operation room to the CT scan and from CT scan to the neurosurgical intensive care unit (ICU). Second routine CT scans were performed at about 24 hours after surgery and allowed follow-up of the volume of intracranial air.

Patients randomized to the treatment arm were ventilated with FiO_2_ = 100% at 1.0 atmosphere absolute (ATA) via the ETT for a period of 3 hours, starting immediately after their admission to the neurosurgical ICU. Anaesthesia was maintained by continuous infusion of propofol. PEEP was adjusted at 5 cm H_2_O. Care was taken to maintain stable mean arterial blood pressure (MABP), heart rate, body temperature, and paCO_2_ to avoid confounding factors that could influence cerebral perfusion pressure. Partial arterial oxygen pressure (PaO_2_) was monitored on-line to screen for signs of pulmonary O_2_ toxicity.

Patients randomized to the control arm were weaned off anaesthesia and then were extubated. Patients in the treatment arm were weaned off and were extubated after completion of normobaric hyperoxia.

ABG was taken and pH, partial oxygen pressure (pO_2_), partial carbon dioxide pressure (pCO_2_), oxygen saturation (SaO_2_), and haemoglobin (Hb) were recorded after surgery and immediately after extubation in all patients.

The neuroradiologist was blinded to treatment and intraoperative patient data. Routine cranial CT scans (GE LightSpeed VFX) were performed with secondary reconstruction of axial slices with 1.25 mm slice thickness. A semi-automated segmentation technique was performed by means of the medical image analysis program YaDiV (Yet Another DICOM Viewer) [15]. Air volume was expressed in cubic millilitres. Epidural air at the craniotomy site was excluded from the measurement.

Two co-primary endpoints were assessed. The first co-primary endpoint was defined as the mean change of air volume (CT1-CT2) in ml. The air resorption rate defined as the mean change of air volume divided by the time between the two CT scans was the second co-primary endpoint.

The Stroop test with two subtasks was performed one day before surgery and 24 hours after surgery [16, 17]. In the first task, patients were requested to name the displayed colour words, in the second task, patients had to name the colour of words instead. The number of failures and the time needed for the task was noted. Furthermore, patients were asked to indicate their level of sleepiness according to the Stanford Sleepiness Scale (SSS) 24 hours after surgery [18]. Sleepiness was defined as both a fundamental state and a tendency to fall asleep [19].

All patients were followed by the study physicians until time of discharge. All patients were observed specifically for any sign or symptom of pulmonary oxygen toxicity such as coughing, substernal discomfort, and dyspnea. Scheduled follow-up at 3 months and 1 year after surgery was performed in all patients.

### Statistical analysis

Baseline characteristics were presented (quantitative parameters: mean, standard deviation (SD), range; qualitative parameters: absolute and relative frequencies) and treatment arms were compared descriptively using two-sided t-tests or χ^2^ tests.

Both primary endpoints were analysed using an analysis of covariance (ANCOVA) including the respective baseline values. A 95% confidence intervals for the treatment effect (control—treatment) and the corresponding p-values was calculated from the ANCOVA model. A two-sided p-value less than 0.05 was termed statistically significant. Estimates of the mean change and respective standard errors (SE) from the ANCOVA model per treatment arm were calculated. Sensitivity analyses included gender and age (dichotomized by the respective median) into the model and the respective subgroups have been reported separately.

Differences in mean time for the Stroop test (postoperative—preoperative) were analyzed in line with the primary endpoints. In addition no failure versus one or more failures in word reading/colour naming were compared between the treatment and control arm using descriptive χ^2^ tests.

The SSS was analysed using descriptive two-sided t-test and Fisher’s exact test.

Blood gas parameters were only explored in line with baseline characteristics.

Sample size had been calculated based on the study by Gore et al [20]. In this study, 6 patients, who had normobaric hyperoxia at FiO_2_ 68%, over a non-rebreather mask for a period of 24 hours, were compared to 7 patients who were breathing room air. Assuming a two-sided type I error of 5%, a power of 90%, for the mean change of pneumocephalus a total of 22 patients per treatment arm (difference:16.6, common SD: 16.3) and for the mean air absorption rate (difference:1.37, common SD: 0.96) a total of 12 patients per treatment arm were considered necessary. Subsequently, 44 patients were planned for recruitment.

## Results

### Patient’s characteristics

**Fig 1:**
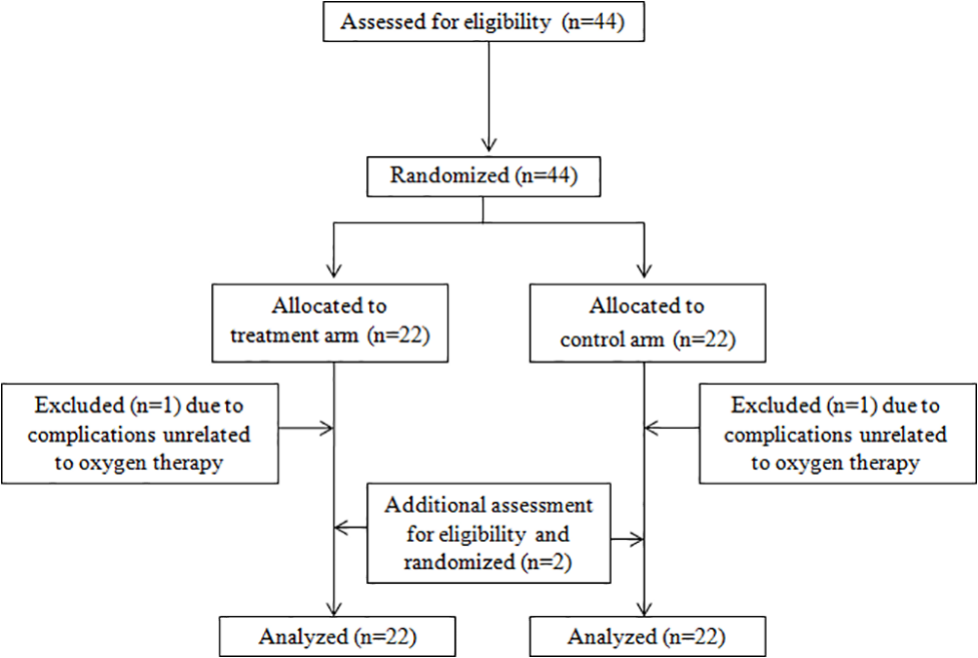
Trial profile.

Originally, 22 patients were planned to be included into each treatment arm. Two patients (one in each arm, both male, 26 and 37 years old) showed unforeseen postoperative complications unrelated to oxygen therapy after randomization and thus were excluded from the statistical analyses. To maintain the statistical power, two additional patients were included after additional approval from the local ethics committee had been obtained (Fig 1).

A total of 44 patients, 8 men and 14 women in the treatment arm and 11 men and 11 women in the control arm, were evaluated. No evidence for differences between treatment groups was observed for age, gender, weight, and height (Table 1).

**Table 1:** Baseline characteristics of the study population.

Variables	Treatment arm (n = 22)	Control arm (n = 22)	p*
Age (years)			0.64^1^
Mean ± SD	52.95 ± 14.09	50.91 ± 14.83	
Range	25–75	23–80	
Sex, n (%)			0.35^2^
Male	8 (36.4)	11 (50.0)	
Female	14 (63.6)	11 (50.0)	
Height (cm)			0.56^1^
Mean ± SD	170 ± 12.17	171.82 ±8.07	
Range	154–194	161–190	
Weight (kg)			0.21^1^
Mean ± SD	85.68 ± 14.88	79.14 ± 18.99	
Range	62–120	47–135	

SD = standard deviation

* p<0.05 = significant

^1^ two-sided t-test

^2^ χ^2^-test

Tumor size and the distribution of pathological diagnoses did not differ significantly between the two groups. Histopathological diagnosis in the treatment arm was vestibular schwannoma (n = 6), meningioma (n = 7), ependymoma (n = 2), aneurysm (n = 1), lipoma (n = 1), hemangioblastoma (n = 1), arachnoidal cyst (n = 1), epidermoid cyst (n = 1), and astrocytoma (n = 2). Patients in the control arm had vestibular schwannoma (n = 11), cavernous hemangioma (n = 2), meningioma (n = 2), subependymoma (n = 1), astrocytoma (n = 1), pineal cyst (n = 1), hemangioblastoma (n = 2), and epidermoid cyst (n = 1).

Table 2 reports data of explorative ABG analyses, which were examined after closure of craniotomy (approximately 30 minutes before the end of the surgery), and immediately after extubation. No differences were observed between the treatment arms.

**Table 2:** Explorative arterial blood gas (ABG) analysis, obtained after closure of the craniotomy, and immediately after extubation.

Variables	after closure of the craniotomy			immediately after extubation		
	Treatment arm (n = 22)	Control arm (n = 22)	p*	Treatment arm (n = 22)	Control arm (n = 22)	p*
pH			0.91			0.66
Mean ± SD	7.42 ± 0.04	7.42 ± 0.04		7.39 ± 0.04	7.38 ± 0.07	
Range	7.32–7.48	7.35–7.48		7.31–7.48	7.19–7.57	
pO_2_ (mmHg)			0.15			0.50
Mean ± SD	204.12 ± 94.25	248.85 ± 109.51		144.91 ± 85.62	129.08 ± 69.90	
Range	109.60–572.00	137.00–492.80		81.30–399.90	53.00–395.60	
pCO_2_ (mmHg)			0.92			0.52
Mean ± SD	38.90 ± 4.52	38.75 ± 4.89		43.30 ± 6.39	42.65 ± 6.89	
Range	26.00–46.50	25.40–47.60		32.00–59.60	24.80–58.00	
O_2_ saturation (%)			0.36			0.37
Mean ± SD	99.22 ± 0.37	99.32 ± 0.38		98.21 ± 1.45	97.63 ± 2.60	
Range	98.50–100.00	98.50–100.00		94.60–99.80	87.00–100.00	
Hb (g/100 ml)			0.56			0.99
Mean ± SD	11.76 ± 1.71	12.04 ± 1.38		12.53 ± 1.69	12.53 ± 1.658	
Range	8.30–16.50	9.20–14.30		8.7–16.1	9.6–15.9	

Hb = haemoglobin; SD = standard deviation

* p<0.05 = significant; two-sided t-test

The distribution of pneumocephalus was comparable in both groups after surgery. Mean baseline volume of pneumocephalus was 109.09 ± 59.46 ml in patients in the treatment arm and 104.7 ± 62.66 ml in patients in the control arm.

### Changes of intracranial air volume and air resorption rate

**Fig 2:**
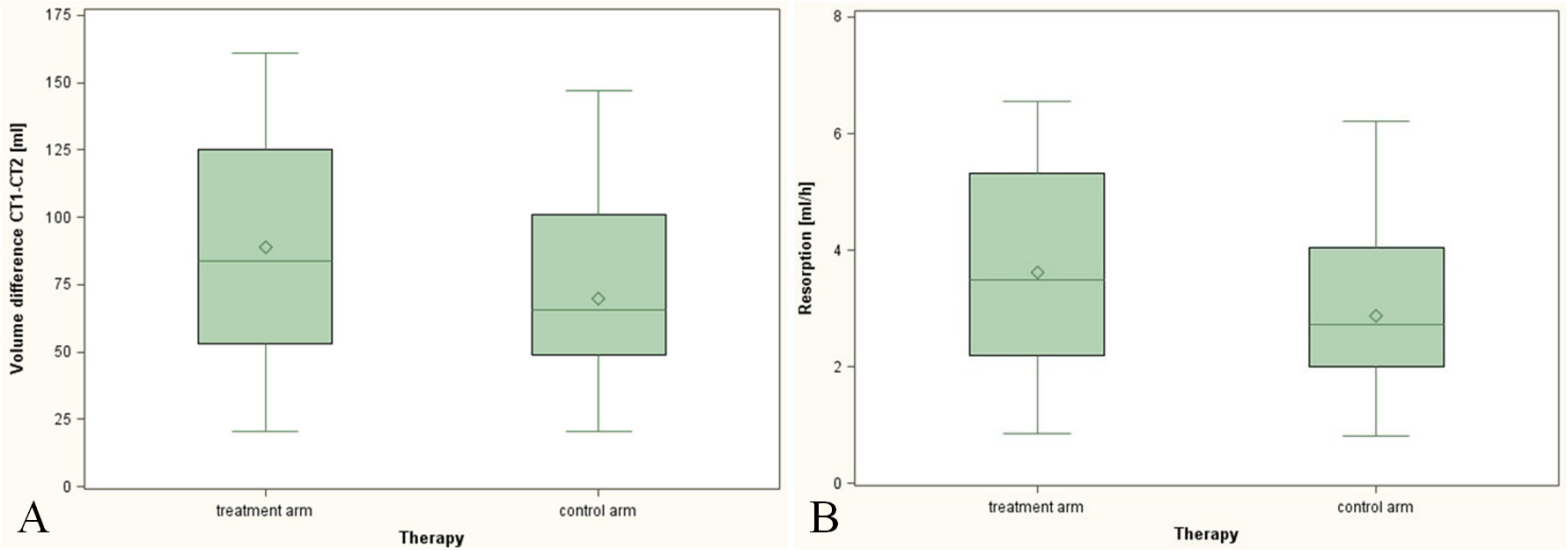
Box plots for the mean volume change of pneumocephalus (A) and air resorption rate (B) between CT1 and CT2.

Changes in volume of pneumocephalus (A) and air resorption rates (B) in both arms are shown in Fig 2. Following the administration of normobaric hyperoxia, the first co-primary endpoint mean volume change of pneumocephalus was 87.83 (SE: 3.31) ml in patients treated with 100% O_2_ and it was 71.29 (SE: 3.31) ml in patients in the control arm (ANCOVA; estimate: 16.54 ml; 95% confidence interval (CI): [-26.00–7.08]; p = 0.001; Table 3). The second co-primary endpoint air resorption rate was higher as well in patients treated with 100% O_2_ (3.57; SE: 0.13 ml/h) as compared to untreated patients (2.93; SE: 0.13 ml/h; ANCOVA; estimate: -0.63; 95% CI: [-1.01, -0.26]; p = 0.0015).

**Table 3:** Changes of intracranial air volume and air resorption rate (control—treatment) [ml].

Variables	Treatment arm	Control arm	Difference	95% confidence interval		p*
	Mean (SE); (n)	Mean (SE); (n)	Mean	Diff.	Vol.	
Difference Vol.[ml]	87.83 (3.31); (22)	71.29 (3.31); (22)	-16.54	-26	-7.08	0.001
Resorption rate[ml/h]	3.57 (0.13); (22)	2.93 (0.13); (22)	-0.63	-1.01	-0.26	0.0015

SE = standard error

* p<0.05 = significant; ANCOVA

Subgroup analyses revealed that mean volume change is more pronounced in men than in women and more distinct in older than in younger patients. In subgroups formed by age and gender, and adjusting for baseline volume (Table 4), a mean difference of volume change of -32.92 (ANCOVA; 95% CI: [-50.94, -14.90]; p<0.001) in older men was observed. The respective treatment effect in older women was -16.79 (ANCOVA; 95% CI: [-33.51, -0.08]; p = 0.049). The respective treatment effects were -3.45 (ANCOVA; 95% CI: [-23.91, 17.03]; p = 0.73) in younger men and -7.63 in younger women (ANCOVA; 95% CI: [-23.99, 8.73]; p = 0.35). Subgroup analyses for the air resorption rate supported also the primary analysis. In line with the subgroup analyses for the mean volume change, the largest treatment effect was observed for older men (Table 5).

**Table 4:** Results of sensitivity analysis for mean change of pneumocephalus volume (control—treatment) [ml].

Variables	Treatment arm	Control arm	Diff. vol.	95% confidence interval		p*
	Mean (SE); (n)	Mean (SE); (n)	Mean	Diff.	Vol.	
Age ≤52.2 years						
Male	79.73 (8.05); (3)	76.28 (6.18); (5)	-3.45	-23.91	17.03	0.73
Female	83.33 (4.89); (8)	75.70 (6.32); (5)	-7.63	-23.99	8.73	0.35
Age >52.2 years						
Male	86.39 (6.16); (5)	53.47 (6.33); (6)	-32.92	-50.94	-14.90	<0.001
Female	98.56 (5.64); (6)	81.78 (5.85); (6)	-16.79	-33.51	-0.08	0.049

SE = standard error

* p<0.05 = significant; ANCOVA

**Table 5:** Results of sensitivity analysis for air resorption rate (control—treatment) [ml/h].

Variables	Treatment arm	Control arm	Diff. vol.	95% confidence interval		p*
	Mean (SE); (n)	Mean (SE); (n)	Mean	Diff.	Vol.	
Age ≤52.2 years						
Male	3.12 (0.34); (3)	3.06 (0.26); (5)	-0.06	-0.93	0.80	0.89
Female	3.44 (0.21); (8)	3.10 (0.27); (5)	-0.34	-1.08	0.34	0.32
Age >52.2 years						
Male	3.62 (0.26); (5)	2.34 (0.27); (6)	-1.27	-0.51	-2.03	0.002
Female	3.89 (0.24); (6)	3.29 (0.25); (6)	-0.60	-1.30	0.11	0.094

SE = standard error

* p<0.05 = significant; ANCOVA

We did not correlate the amount of intracranial air with the patient’s cranial volume. However, cranial sizes among the study patients did not differ markedly.

### Stroop test

Summarized results of preoperative and postoperative Stroop tests are shown in Table 6. One patient in the treatment arm and four patients in the control arm were not able to perform the postoperative Stroop test completely. No differences between preoperative and postoperative measurements were found in the remainder. In addition, there were no differences between the treatment arm and the control arm.

**Table 6:** Results of Stroop test of patients in treatment and control arm.

Variables	Treatment arm	Control arm	Diff. means	95% confidence interval		p*
	Mean (SE); (n)	Mean (SE); (n)	Control—Treatment	Limits Diff	Mean	
***Differences***: *(Postoperative—Preoperative)*						
Failure in words reading (Task 1)	0.897 (0.507); (21)	1.272 (0.534); (19)	0.37	-1.11	1.87	0.62
Mean time ± SD (seconds)	20.72 (6.81); (21)	26.40 (7.17); (19)	5.67	-14.48	25.83	0.57
Failure in color naming (Task 2)	0.97.72 (0.60); (21)	1.64 (0.64); (18)	0.67	-1.13	2.47	0.45
Mean time ± SD (seconds)	19.32 (7.89); (21)	25.65 (8.52); (18)	6.32	-17.24	29.89	0.59

SD = standard deviation

* p<0.05 = significant; ANCOVA

### Stanford Sleepiness Scale (SSS)

Table 7 shows the distribution of patients according to their SSS score. There was a difference between treatment and control arm on the basis of the categorical data (Fisher’s exact test: p<0.001). Patients in the treatment were more alert, with a lower score of 3.18 ± 0.3, in comparison to patients in the control arm (mean 4.27 ± 0.3; t-test; 95% CI: [0.22, 1.98]; p = 0.015).

**Table 7:** Distribution of Stanford Sleepiness Scale at 24 hours after surgery.

Scale	Treatment arm, n (%)	Control arm, n (%)	p*
			<0.001
1	1 (4.5)	0 (0.0)	
2	8 (36.4)	2 (9.1)	
3	6 (27.3)	6 (27.3)	
4	3 (13.6)	4 (18.2)	
5	1 (4.56)	4 (18.2)	
6	3 (13.6)	6 (27.3)	
7	0 (0.0)	0 (0.0)	

* p<0.05 = significant; two-sided Fisher’s exact test

## Discussion

The main finding of our study is that patients who were treated with normobaric hyperoxia at FiO_2_ 100% via ETT for 3 hours showed a significantly higher resorption rate of postoperative pneumocephalus as compared to untreated patients.

In 2008, Gore et al. demonstrated a significant increase of pneumocephalus resorption in 6 patients, who had normobaric hyperoxia at FiO_2_ 68% over a non-rebreather mask for a period of 24 hours, resulting in a mean air volume reduction of 65%, in comparison to 7 patients who were breathing room air with a mean air volume reduction of 31%. The mean air volume change in the treated patients was 35 ml and the mean air absorption rate was 1.26 ml/h [20]. Our results show even higher rates both for mean air volume change (87.83 ± 3.31 ml) and for air resorption rate (3.57 ± 0.13 ml/h) after administration of normobaric hyperoxia. These differences are most likely explained by the mode and by the amount of delivered O_2_, indicating that O_2_ delivery at higher FiO_2_ over ETT is more effective to treat pneumocephalus.

Delivery of supplemental O_2_ with different non-invasive systems was able to provide a high mean FiO_2_, but systems can potentially lead to inadequate delivery of the prescribed oxygen in some instances [21, 22]. Simple face masks and nasal cannulas do not deliver accurately FiO_2_ because of the inconsistent admixture of O_2_ with room air from leakage and mouth breathing. A non-rebreather mask has small rubber valves, which prevent admixture of CO_2_ and water vapor with the inspired O_2_. Because of gas leakage, such masks deliver an FiO_2_ of at most 80 to 90% [23]. Considering these limitations, we decided to take advantage of ETT in our study to ensure precise delivery of FiO_2_.

As posterior fossa surgery may be complicated by lower cranial nerve palsies, careful assessment of lower cranial nerve function is essential before extubation. The intubation period in patients after posterior fossa surgery is often longer than in other neurosurgical procedures. For the purpose of the study, the intubation period in patients in the treatment arm was prolonged for applying normobaric hyperoxia.

There are several concerns about the safety of administration of 100% O_2_ for a longer period of time. The development of oxygen toxicity depends both on the partial pressure of O_2_ and the duration of administration. Pulmonary damage develops in the form of diffuse alveolar haemorrhage and interstitial oedema, as well as pulmonary infection, particularly if the lungs were exposed to a high O_2_ partial pressure for more than 48 hours at 1.0 ATA [24, 25]. Healthy people who inspire 100% O_2_ for up to 12 hours do not show pulmonary abnormalities [26–28].

The term unit pulmonary toxicity dose (UPTD) was introduced by Bardin & Lambertsen, in order to quantitate relative O_2_ doses. One UPTD corresponds to 1 minute exposure of 100% O_2_ at 1.0 ATA [29]. The recommended O_2_ exposure in a single treatment is under 615 UPTD, whereby the extreme upper limit is 1425 UPTD. The administration of 100% O_2_ for 3 hours in our study corresponded approximately to 180 UPTD ensuring its safety.

In this study no complications were observed that are likely to be attributable to normobaric hyperoxia. ABG levels in patients treated with normobaric hyperoxia were within acceptable levels, and did not differ significantly from levels in the control arm. Thus, we didn’t see any problems of administration of normobaric hyperoxia at FiO_2_ 100% for 3 hours for the treatment of pneumocephalus.

Compression of the frontal lobes is the most common finding in radiological studies of pneumocephalus [30, 31]. We used the Stroop test in our study since it is a common measure of frontal lobe function and easy to apply [32]. Although no statistically significant differences were found between groups in our study, it is of interest to note that only one patient in the treatment arms was unable to complete the Stroop test postoperatively as compared to 4 patients in the control arm.

The effect of normobaric hyperoxia on the recovery of sleepiness due to pneumocephalus 24 hours after surgery was more pronounced in patients in the treatment arm. There was a trend towards that patients in the treatment arm did recovery earlier than in the control arm.

The strengths of our study is its prospective design, minimizing systematic bias in treatment by the randomization process, and minimizing observer bias for the primary endpoints by masking the assigned treatment to the radiologist and to the patient. Furthermore, this study was based on a pre-existing plausible hypothesis that normobaric oxygen would be effective to treat pneumocephalus which is now confirmed in this study.

One limitation of this trial was that the secondary endpoints were not assessed in an observer-blind fashion due to practical reasons. We acknowledge, that the Stroop test and the SSS may not be sensitive enough to detect subtle attention and alertness deficits, and also that there are no validated studies with these scales. Despite its limitations the present study provides valid data about the effectiveness of normobaric hyperoxia at FiO_2_ 100% via ETT for the treatment of space-occupying postoperative pneumocephalus.

In conclusion, our results demonstrate a significantly higher reduction in pneumocephalus volume and a higher resorption rate of postoperative air with normobaric hyperoxia. Oxygen administration as performed in our study is a safe, simple, and technically and clinically feasible method. We suggest that oxygen therapy might be rationally applied in the future management of pneumocephalus after a surgical intervention in the semisitting position that should be administered in the elderly patients and has demonstrated largest effects in older men.

## Supporting Information

S1 CONSORT Checklist CONSORT Checklist. (DOC)

S1 File (XLS)

S2 File (PDF)

S1 Protocol Trial Protocol. (DOC)

S2 Protocol Trial Protocol in English. (DOC)
