# Supplementary material for: Normobaric Hyperoxia for Treatment of Pneumocephalus after Posterior Fossa Surgery in the Semisitting Position: A Prospective Randomized Controlled Trial
Source: PLoS One. 2015 May 20;10(5):e0125710. doi: 10.1371/journal.pone.0125710 (PMC4439020; doi:10.1371/journal.pone.0125710)
Supplement: S2 File — (PDF) [file pone.0125710.s003.pdf]

Statistical Analysis Report

Version 1.2

Protocol Title:
“Prospektive randomisierte Studie zur Analyse der Wirkung von 100% reinem normobarem Sauerstoff zur Behandlung des postoperativen Pneumozephalus nach neurochirurgischen Eingriffen in halbsitzender Position.”

Date 15.11.2013

Frank Biertz **Anika Großhennig**

....

Table of Contents:

Table of Contents: 2
1. Introduction 3
2. Endpoints 3
   2.1. Primary Endpoints 3
   2.2. Secondary Endpoints 4
   2.3. Exploratory Endpoints 4
3. Definition of variables 4
4. Randomization/ Blinding 5
5. Analysis cohorts 5
   5.1. Safety cohort 5
   5.3 ITT cohort 6
   5.4 PP cohort 6
6. Statistical methods 6
   6.1 Deskriptive Auswertung der Ausgangswerte 6
   6.2 Primäre Auswertung 6
   6.3 Sensitivitätsanalysen 6
   6.4 Sekundäre Auswertung 7
   6.6 Missing data 7
7. Statistical analyses 7
   7.1. Patient disposition 7
      7.1.1 Screening Failures 7
      7.1.2 Patient accrual by sites 7
   7.2. Eligibility, demographic and baseline characteristics 7
      7.2.1 Eligibility criteria 7
      7.2.2 Demographic and baseline characteristics 8
   7.3. Primary analysis 11
      7.3.1 Confirmatory analysis 12
      7.3.2 Sensitivitätsanalyse 13
   7.4 Secondary analysis 15
   7.4.1 Stanford Sleepiness Scale 15
   7.4.2 Farbe-Wort-Interferenz-Test (Stroop) 15
8. Appendix 17

1. Introduction

Es wurde eine prospektive randomisierte Studie zur Analyse der Wirkung von 100% reinem normobarem Sauerstoff zur Behandlung des postoperativen Pneumozephalus nach neurochirurgischen Eingriffen in halbsitzender Position durchgeführt.

Das primäre Ziel dieser prospektiven, kontrollierten randomisierten Studie ist es zu überprüfen, ob die Absorption des postoperativen Pneumozephalus nach Eingriffen im Bereich der hinteren Schädelgrube in halbsitzender Lagerung durch die zusätzliche Beatmung mit normobarem 100%tigem Sauerstoff beschleunigt wird. Hierfür sollen 44 Patienten zwischen 18 und 80 Jahren in zwei Gruppen randomisiert werden. Die Therapiegruppe soll postoperativ 100%tigen reinen normobaren Sauerstoff über einen endotrachealen Tubus erhalten. Die Kontrollgruppe wird eine Beatmung mit der üblichen O2-Konzentration erhalten.

Die Studie wurde mit zwei co-primären Endpunkten geplant. Zum einen soll die Veränderung des Luftvolumens im Kopf betrachtet werden. Hierfür wird die Differenz zwischen den Volumina 24 Stunden nach dem neurochirurgischen Eingriff und direkt postoperativ bestimmt. Der zweite co-primäre Endpunkt ist die Absorptionsrate pro Stunde. Nur wenn die Volumenabnahme und die Absorptionsrate in der Therapiegruppe höher sind, ist gezeigt, dass die Sauerstofftherapie der Standardbehandlung überlegen ist.

In den sekundären Analysen soll zusätzlich untersucht werden, ob sich Therapie- und Kontrollgruppe bezüglich Prozessen bei denen das Ventrikelsystem geöffnet wird von denen unterscheiden bei denen das Ventrikelsystem[1] nicht geöffnet wird. Ferner soll geklärt werden, ob sich für die Patienten der beiden Therapiearme Unterschiede der Konzentrationsfähigkeit, gemessen am Farbe-Wort-Interferenztest, ergeben. Darüber hinaus soll die Selbsteinschätzung des Grades der Schläfrigkeit der Patienten (Stanford Sleepiness Scale) zwischen Therapie- und Kontrollgruppe miteinander verglichen werden.

Die primäre Hypothese dieser prospektiven, kontrollierten randomisierten Studie ist, dass die Absorption bei Patienten mit Sauerstofftherapie innerhalb von 24 Stunden nach dem operativem Eingriff bereits zu einer größeren Volumenveränderung und einer höheren Absorptionsrate führt als bei Patienten, die eine Beatmung mit einer Standard-Sauerstoffkonzentration erhalten. Die Auswertung basiert auf dem Datensatz der von Herrn Dr. Hong am 02.11.2012 der Biometrie zur Verfügung gestellt wurde. Die Daten wurden anschließend geprüft und es waren in Absprache mit Herrn Hong einige Ergänzungen erforderlich.

2. Endpoints

2.1. Primary Endpoints

Zeitnah nach dem operativen Eingriff wird das Volumen des Pneumozephalus mittels einer CT-Untersuchung ermittelt (Vol-CT1 [ml]). Wenn der Patient alle Voraussetzungen für den Studieneinschluss erfüllt, erfolgt eine randomisierte Zuteilung von postoperativer Beatmung mit 100%-tigem O2- oder normalem O2-Anteil. Die Beatmung mit 100%-Sauerstoff erfolgt für maximal 3 Stunden, wenn eine weitere Beatmung erforderlich ist, erfolgt diese, wie in der Gruppe mit Standardtherapie mit normalen O2-Anteil. 24 Stunden nach der ersten CT-Aufnahme erfolgt eine zweite CT-Untersuchung und eine Volumenbestimmung des Pneumozephalus (Vol-CT2 [ml]). Es ergibt sich hier eine Abweichung von der im Studienprotokoll hierzu beschriebenen Vorgehensweise. Das Studienprotokoll führt aus, dass die zweite CT-Messung des Pneumocephalus-Volumens 24 Stunden nach der Extubation erfolgen soll. Andererseits wird die 2. CT-Messung jedoch auch terminiert auf 24 Stunden nach der Operation beschrieben. Die Zeitpunkte der Extubation sind nur nach klinischen Aspekten durch den Anästhesisten zu bestimmen und wie die Daten zeigen, deutlich verschieden für die einzelnen Patienten. Die einzigen Zeitpunkte zu denen die Volumina des Pneumocephalus bekannt sind, ist der Zeitpunkt der ersten und der zweiten Computertomografie. Wie die Datenlage zeigt ist die Verteilung der Zeit zwischen CT1 und CT2 für beide Gruppen ähnlich und der Mittelwert nur wenig verschieden von 24 Stunden (100% O2/ Standard, Mean:24,82 / 24,48 [h]; Median:24,53 / 24,16; [h]). Der radiologische

1 Informationen über Eröffnung des Ventrikelsystems , telefonisch erhalten „ für alle Patienten gleiche Art der Eröffnung"

Untersucher war verblindet gegenüber der angewandten Therapie. Entgegen der Planung im Studienprotokoll, konnten die STROOP-Test hingegen nicht verblindet durchgeführt werden,

Präzise sind die co-primären Endpunkte wie folgt definiert:

- Die Volumendifferenz des Pneumozephalus zum ersten Zeitpunkt abzüglich des Volumens zum zweiten Zeitpunkt (Vol-CT1 – Vol-CT2 [ml])
- Der zweite co-primäre Endpunkt ist die Absorption pro Stunde in [ml/h], berechnet als Vol-(CT1 – Vol-CT2) / Diff-Time-CT2-CT1 (Zeitabstand zwischen den beiden CT-Untersuchungen umgerechnet in Stunden).

2.2. Secondary Endpoints

Es wird gemessen, ob die Konzentrationsfähigkeit der Patienten gemessen am Farbe-Wort-Interferenztest für die beiden Therapiegruppen unterschiedlich ist. Zusätzlich wird die Selbsteinschätzung des Patienten zur Schläfrigkeit anhand der Stanford Sleepines Scale verglichen. Die Werte der postoperativen Blutgasanalyse werden auf deutliche Unterschiede für die beiden Therapiegruppen untersucht.

2.3. Exploratory Endpoints

n.a.

3. Definition of variables

- SEX Geschlecht: weiblich / männlich
- AGE Alter in Jahren
- HEIGHT Größe in cm.
- Weight Gewicht in kg
- Diff-Time-CT2-CT1 Abstand zwischen den CT-Untersuchen zum Zeitpunkt 1 und 2 umgerechnet in Stunden
- Vol-CT1 Volumen des Pneumozephalus zum Zeitpunkt 1 in ml
- Vol-CT2 Volumen des Pneumozephalus zum Zeitpunkt 2 in ml
- Diff-Volume-CT1-CT2 Volumendifferenz des Pneumozephalus zum Zeitpunkt 1 abzüglich zum Zeitpunkt 2
- Absorption des Pneumozephalus-Volumens in ml/ h. wird bestimmt aus
  Diff-Volume-CT1-CT2 / Diff-Time-CT2-CT1 [ml / h]
- SSS "Stanford Sleepiness Scale" in den Kategorien 1 bis 7. (Einzelheiten s. Anlage II)

Blutgasanalysen:

Zeitpunkt 1: Intraoperativ nach Duraverschluss, vor der Anwendung der Beatmung mit der experimentellen bzw. standardisierten O2-Konzentration.

- BGA_1_PH_num PH-Wert
  BGA_1_PO2_num Sauerstoffpartialdruck im Blut in mmHg
- BGA_1_PCO2_num CO2-Partialdruck in mmHg
- BGA_1_O2SAT_num Sauerstoffsättigung in %
- BGA_1_BE_num Base Excess in mmol/l
- BGA_1_HB_num Hämoglobin in g/100 ml

-

Zeitpunkt 2: Nach der Extubation bzw. am Ende der 100% O2-Beatmung

BGA_2_PH_num	PH-Wert
BGA_2_PO2_num	Sauerstoffpartialdruck im Blut in mmHg
• BGA_2_PCO2_num	CO2-Partialdruck in mmHg
• BGA_2_O2SAT_num	Sauerstoffsättigung in %
• BGA_2_BE_num	Base Excess in mmol/l
BGA_2_HB_num	Hämoglobin in g/100 ml

Stroop-Test / Farbe-Wort-Interferenztest

Messung der Konzentrationsfähigkeit erfolgt zu 2 Zeitpunkten und besteht aus dem Wortnamen- und dem Farbnamen-Test. Es wird die Anzahl der Fehler und die benötigte Zeit für die Durchführung des Testes in Sekunden ermittelt (Einzelheiten s. Anlage I).

Die erste Messung erfolgt präoperativ, als Baseline-Bestimmung

- Praeop1_wort_fehl — Anzahl der Fehler im Wortnamen-Test
- Praeop1_wort_time — Benötigte Zeit in Sekunden für den Wortnamen-Test
- Praeop2_farbe_fehl — Anzahl der Fehler im Farbnamen-Test
- Praeop2_farbe_time — Benötigte Zeit in Sekunden für den Farbnamen-Test

Die zweite Messung erfolgt postoperativ, zeitnah zur Durchführung der zweiten CT-Untersuchung.

- Postop1_wort_fehl — Anzahl der Fehler im Wortnamen-Test
- Postop1_wort_time — Benötigte Zeit in Sekunden für den Wortnamen-Test
- Postop2_farbe_fehl — Anzahl der Fehler im Farbnamen-Test
- Postop2_farbe_time — Benötigte Zeit in Sekunden für den Farbnamen-Test

Die Analyse erfolgt mit den Differenzwerten jeweils für die Anzahl der Fehler und die benötigte Zeit für den Test im Wortnamen-Test und im Farbnamen-Test. Die Differenz wird gebildet aus den postoperativen Werten minus der prä-operativen Werte.

4. Randomization/ Blinding

Da es sich um eine offene Studie handelt, ist die Randomisierung zentral an unabhängiger Stelle im Institut für Biometrie als Telefonrandomisiserung durchgeführt worden. Dabei wurde der Patient zuerst auf seine Einschlussfähigkeit untersucht, und, falls die Voraussetzungen für den Studieneinschluss vorliegen, eingeschlossen und anschließend durch das Institut der Biometrie der MHH, in einem Randomisierungsverfahren mit variabler Blocklänge der Therapiegruppe zugeteilt.

Die Befundung der CT-Aufnahmen geschah durch einen unabhängigen Radiologen, der keine Kenntnis über die zugeteilte Therapie hat.

5. Analysis cohorts

5.1. Safety cohort

n.a.

5.2. Changes to the planned conduct

Aus den ursprünglich geplanten 44 Patienten konnte bei 2 Patienten, aufgrund einer unerwarteten Komplikation während des postoperativen stationären Aufenthaltes auf der Intensivstation und zusätzlich für einen der beiden Patienten aus organisatorischen Gründen, keine zweite postoperative CT-Kontrolle durchgeführt werden, so dass die Studie bei diesen 2 Patienten abgebrochen wurde. Es liegen für beide Patienten keine Messungen vor, die eine Bestimmung der primären und sekundären Endpunkte ermöglichen würden.

. Da in der ursprünglichen Fallzahlplanung keine Dropouts berücksichtigt wurden,r wurde eine Fallzahlerhöhung für 2 Patienten beantragt. Diesem Antrag wurde von der Ethikkommission entsprochen. .

5.3 ITT cohort

Der Auswerteplan sah die Analyse nach ITT vor, wobei alle Patienten für die Gruppe der zugeteilten Therapie ausgewertet werden und fehlende Werte so ersetzt werden sollten, dass eine konservative Auswertung durchgeführt würde. Für 2 der Patienten waren die erste und die zweite Messungen zur Bestimmung des Endpunktes nicht vorhanden, sodass für diese Patienten kein Endpunkt ermittelt werden konnte Wie unter 5.2 beschrieben, wurde die geplante Fallzahl von 44 beibehalten, in dem für die beiden Patienten, für die der Endpunkt nicht ermittelt werden konnte, die Randomisierung, nach Genehmigung durch die Ethikkommission, für 2 weitere Patient durchgeführt wurde.

5.4 PP cohort

Die Analyse erfolgte per-Protocoll für alle 44 Patienten, die alle Einschlussvoraussetzungen erfüllen, nach der zugeteilten Therapie behandelt wurden und zusätzlich der primäre Endpunkt ermittelbar war.

6. Statistical methods

6.1 Deskriptive Auswertung der Ausgangswerte

Alle deskriptiven Auswertungen werden für die beiden Therapiearme und die Gesamtpopulation durchgeführt. Für qualitative Parameter werden absolute und relative Häufigkeiten, sowie Chi-Quadrat-Tests oder exakte Tests nach Fisher berechnet. Für quantitative Parameter werden Mittelwert (Mean), Standardabweichung (Std), Minimum (Min), Median und Maximum (Max) angegeben. Die beiden Therapien werden mittels zweiseitiger deskriptiver t-Tests miteinander verglichen.

6.2 Primäre Auswertung

Die beiden primären Endpunkte werden jeweils mit einer Kovarianzanalyse (ANCOVA) untersucht. Die abhängige Variable ist der jeweilige Endpunkt. Ferner werden die Behandlung (Standard % Sauerstoff – 100% Sauerstoff [2]) und der Wert des primären Endpunktes direkt nach der Operation in das Modell aufgenommen. Eine größere Volumenänderung unter 100% Sauerstofftherapie wird geschlussfolgert, wenn die obere Grenze des 95% Konfidenzintervalls des Behandlungseffekts kleiner als 0 ist. Eine höhere Absorptionsrate unter 100% Sauerstofftherapie wird gefolgert, wenn die obere Grenze des 95% Konfidenzintervalls des Behandlungseffekts kleiner als 0 ist1. Nur wenn für beide Endpunkte eine Überlegenheit der 100% Sauerstofftherapie gezeigt werden kann, ist der Schluss, dass die Therapie dem bisherigen Standard überlegen ist, zulässig

6.3 Sensitivitätsanalysen

Als Sensitivitätsanalysen wurden die Behandlungseffekte adjustiert für Geschlecht und Alter betrachtet. Hierfür wurde das Alter anhand des Medians dichotomisiert. Für beide primären Endpunkte wurden jeweils drei weitere ANCOVAS berechnet: i) Behandlung, Ausgangswert, Geschlecht; ii) Behandlung, Ausgangswert, Alter dichotomisiert; iii) Behandlung, Ausgangswert, Geschlecht, Alter dichotomisiert.

---

[2]Dieses Vorgehen führt zu den gleichen Schlussfolgerungen wie im Studienprotokoll. Dort wurde geplant die Differenz andersherum (100% Sauerstoff – 21% Sauerstoff) zu berechnen und die unteren Grenzen der Konfidenzintervalle zur Entscheidung heranzuziehen.

6.4 Sekundäre Auswertung

Alle sekundären Auswertungen sind explorativ. Für die Standford Sleepiness Scale nach der OP wzurde ein exakter Test nach Fisher durchgeführt. . Die Anzahl der Fehler im Stroop-Test und die benötigte Zeit im Stroop-Test (jeweils für Wortnamen- und Farbnamentest) werden entsprechend der primären Analyse mit einer ANCOVA ausgewertet. Hierfür werden die Differenzen aus den Werten präoperativ und 24 h postoperativ gebildet und als abhängige Variable modelliert. Als unabhängige Variablen werden Behandlung (Standard % Sauerstoff – 100% Sauerstoff[3]) und der prä OP Wert betrachtet.

Zwischenauswertungen waren nicht geplant und wurden auch nicht durchgeführt.

6.6 Missing data

Bis auf die unter 5.3 genannten 2 Patienten, die nachrekrutiert werden mussten, war die Erfassung des primären Endpunktes komplett möglich. Je einer der Patienten entfiel auf die O_2-Standard- und auf die 100%-O_2-Therapie.

7. Statistical analyses

7.1. Patient disposition

7.1.1 Screening Failures

Siehe unter 5.3

7.1.2 Patient accrual by sites

Die Rekrutierung erfolgte in einem Zentrum, der Einschluss für den ersten Patienten erfolgte am 30.08.2010. Die Rekrutierung endete am 11.04.2012 mit dem Einschluss des letzten Patienten.

7.2. Eligibility, demographic and baseline characteristics

7.2.1 Eligibility criteria

Die Patienten wurden einer neurochirurgischen Intervention des Schädels in halbsitzender Position unterzogen.

Einschlußkriterien:

- ·Standardmäßige Narkose– sowie Beatmungsverhältnisse, nach im Haus üblicherweiser verwendeter Methode.
- Regelrechter intraoperativer Verlauf ohne Erwartung einer intrakraniellen Komplikation.
- Nachweis eines intrakraniellen Lufteinschlusses von mindestens 30 ml Volumen in der direkten postoperativen CT-Untersuchung (CT1).

Ausschlußkriterien:

- Patienten mit erwarteter Nachbeatmungszeit über 10 Stunden.
- Patienten mit intrathorakalen Auffälligkeiten (Z.n. Operation, Tumor, Entzündung, COPD, angeborene Fehlbildung sowie mit schwerer Herzinsuffizienz).
- Patienten mit sub-/epiduraler sowie subgalealer Drainage.
- Schwangere oder stillende Patienten.

---

[3]Dieses Vorgehen führt zu den gleichen Schlussfolgerungen wie im Studienprotokoll. Dort wurde geplant die Differenz andersherum (100% Sauerstoff – 21% Sauerstoff) zu berechnen und die unteren Grenzen der Konfidenzintervalle zur Entscheidung heranzuziehen.

7.2.2 Demographic and baseline characteristics

Es konnten 44 Patienten, mit den für die Bestimmung des primären Endpunktes erforderlichen Angaben, eingeschlossen werden. Nachfolgend findet sich die Beschreibung der untersuchten Gruppe und deren Eigenschaften in der Aufteilung auf die beiden Therapiearme.

Bestimmung präoperativ		Therapie		N=44	
		100 % O_2	Standard	Gesamt	p-value
Sex N(%)	Female	14(63.6%)	11(50.0%)	25(56.8%)	0.35[1]
	Male	8(36.4%)	11(50.0%)	19(43.2%)	
Age [year]	Mean	52.95	50.91	51.93	0.64[2]
	Std	14.09	14.83	14.33	
	Min	25	23	23	
	Median	53	52.5	52.5	
	Max	75	80	80	
Height [cm]	Mean	170	171.82	170.91	0.56 [2]
	Std	12.17	8.07	10.24	
	Min	154	161	154	
	Median	164	170	168.5	
	Max	194	190	194	
Weight [kg]	Mean	85.68	79.14	82.41	0.21[2]
	Std	14.88	18.99	17.18	
	Min	62	47	47	
	Median	86	77.5	82.5	
	Max	120	135	135	

Tab.: 1 Demografische Beschreibung der Kohorte und der Therapiegruppen (präoperativ).
Test: [1]CHI2, [2]TTEST, [3]Fisher

Es ergeben sich keine Hinweise darauf, dass die beiden Therapiegruppen bezüglich der genannten demographischen Kriterien ungleichmäßig verteilt sind.

Bestimmung intraoperativ		Therapie		N=44	
		100 % O_2	Standard	Gesamt	p-value
PH-Wert 1	Mean	7.42	7.42	7.42	0.91[2]
	Std	0.04	0.04	0.04	
	Min	7.32	7.35	7.32	
	Median	7.43	7.43	7.43	
	Max	7.48	7.48	7.48	
PO_2 1 [mmHg]	Mean	204.12	248.85	226.48	0.15[2]
	Std	94.25	109.51	103.47	
	Min	109.6	137	109.6	
	Median	184.1	214.45	189.7	
	Max	572	492.8	572	
PCO_2 1 [mmHg]	Mean	38.9	38.75	38.82	0.92[2]
	Std	4.52	4.89	4.65	
	Min	26	25.4	25.4	
	Median	38.3	38.8	38.45	
	Max	46.5	47.6	47.6	
O_2Sättigung..1 [%]	Mean	99.22	99.32	99.27	0.36[2]
	Std	0.37	0.38	0.38	
	Min	98.5	98.9	98.5	
	Median	99.2	99.3	99.25	
	Max	100	100	100	
Base-Excess 1 [mmol/l]	Mean	0.76	0.59	0.67	0.76[2]
	Std	2.13	1.63	1.88	
	Min	-3.8	-2.2	-3.8	
	Median	0.75	1.2	1.2	
	Max	4.3	3.5	4.3	
Hämoglobin 1 [g/100 ml]	Mean	11.76	12.04	11.9	0.56[2]
	Std	1.71	1.38	1.55	
	Min	8.3	9.2	8.3	
	Median	11.35	12.25	11.95	
	Max	16.5	14.3	16.5	

Tab.: 2 Baseline für Blutgasanalyse der Kohorte und der Therapiearme

Test: [1]CHI2, [2]TTEST, [3]Fisher

In der basalen Blutgasanalyse ergeben sich für die Therapiegruppen keine Hinweise auf Unterschiede in der Verteilung der Variablen.

Bestimmung präoperativ		Therapie 100 % O_2	Standard	N=44 Gesamt	p-value
Anzahl der Fehler im **Wortnamen-Test** N(%)	0	18(81.8%)	19(86.4%)	37(84.1%)	0.68[1]
	>= 1	4 (18.2%)	3 (13.6%)	6(15.9%)	
Benötigte Zeit in Sekunden für den **Wortnamen-Test**	Mean	32.97	28.84	30.91	0.25[2]
	Std	13.01	10.51	11.87	
	Min	18.23	17.79	17.79	
	Median	29.12	25.68	27.27	
	Max	64.38	68.41	68.41	

Tab.: 3 Baseline für Farbe-Wort-Interferenz-Test (Stroop) der Kohorte und der Therapiearme

Test: [1]CHI2, [2]TTEST, [3]Fisher

Bestimmung präoperativ		Therapie 100 % O_2	Standard	N=44 Gesamt	p-value
Anzahl der Fehler im **Farbnamen-Test**	0	13(50.0%)	13(50.0%)	26(59.1%)	1.0[1]
	>= 1	9(50.0)	9(50.0%)	18(40.9%)	
Benötigte Zeit in Sekunden für den **Farbnamen-Test**	Mean	60.01	59.36	59.69	0.65[2]
	Std	15.34	14.26	14.64	
	Min	42.61	35.67	35.67	
	Median	56.2	57.4	56.99	
	Max	96.85	91	96.85	

Tab.: 4 Baseline für Farbe-Wort-Interferenz-Test (Stroop) der Kohorte und der Therapiearme

Test: [1]CHI2, [2]TTEST, [3]Fisher

Die basal ermittelten Werte des Stroop-Testes zeigen keinen Unterschied für die Anzahl der Fehler im Wortnamen- und im Farbnamen-Test

Postoperativ mit andauernder Beatmung	N=44	100 % O_2	Standard	Gesamt	p-value
CCT_1_Vol [ml]	Mean	109.09	104.7	106.9	0.81[2]
	Std	59.46	62.66	60.41	
	Min	20.6	21.4	20.6	
	Median	99.2	89.25	95.9	
	Max	212.8	248.2	248.2	

Tab.: 5 Ausgangsvolumen des Pneumozephalus vor Therapie im CT 1

Test: [1]CHI2, [2]TTEST, [3]Fisher

Das basale Volumen des Pneumozephalus zeigt keine deutlichen Unterschiede für die beiden Therapiearme.

7.3. Primary analysis

Für jeden Patienten wird die Differenz aus den Volumina direkt nach der OP und 24 Stunden nach der Extubation gebildet (Diff-Volume-CT1-CT2 in ml).

Zusätzlich wird die Absorption des Luftvolumens pro Stunde für jeden Patienten ermittelt (Diff-Volume-CT1-CT2 / Diff-Time-CT2-CT1) in ml/h.

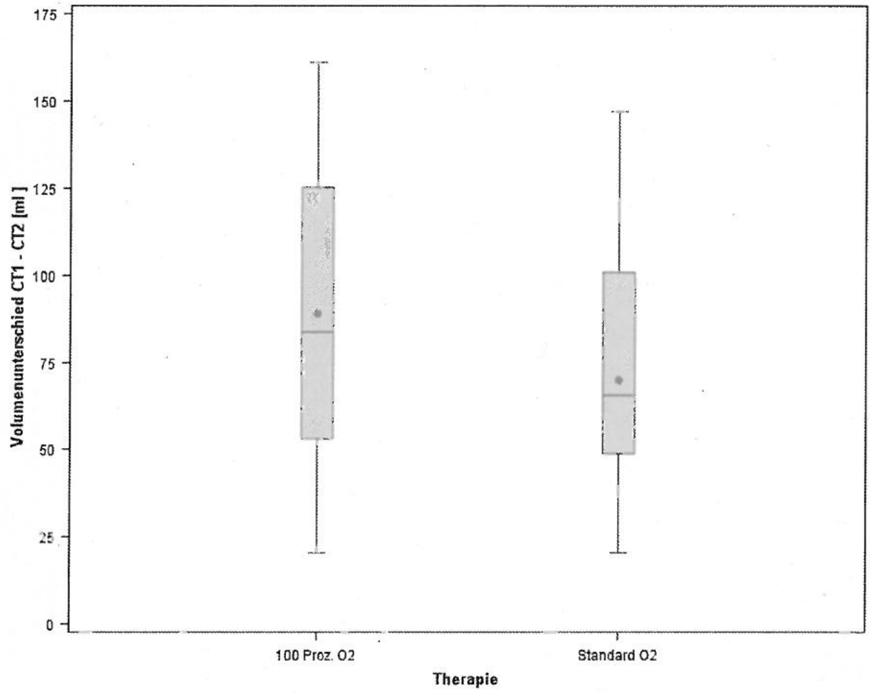

Fig.: 1 Volumendifferenz [ml] zwischen CT1 – CT2 für die beiden Therapiearme

Bei univariater Betrachtung der Volumendifferenz [ml] zeigen sich Unterschiede im Mittelwert, aber auch in der Verteilung der Daten in den beiden Therapiegruppen.

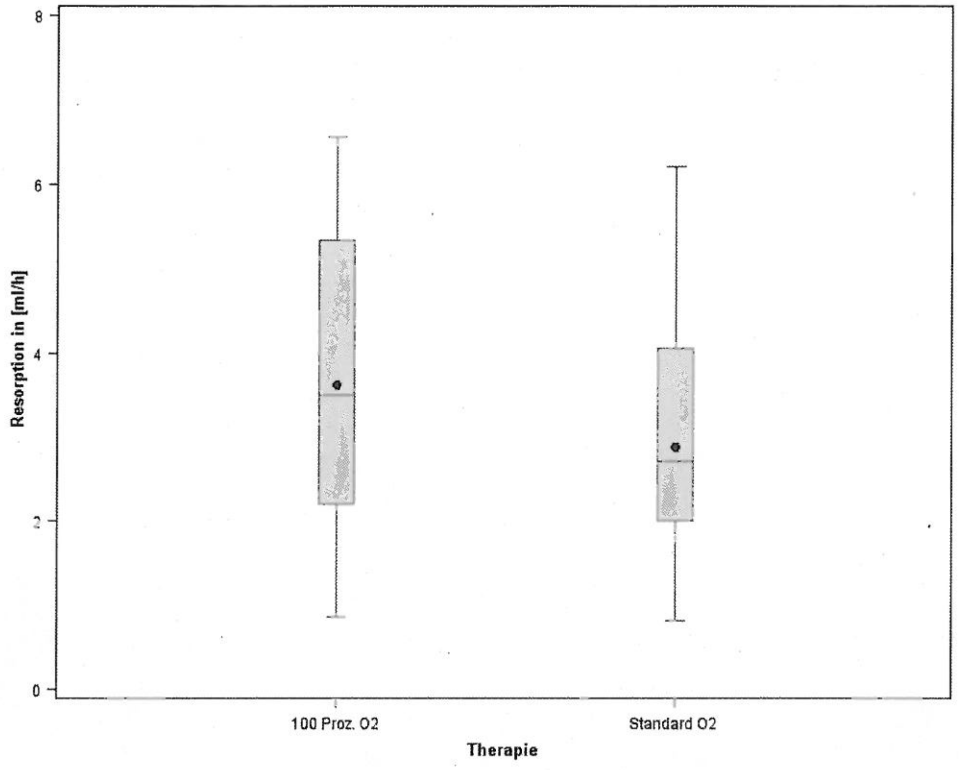

Fig.: 2 Absorption pro h für die beiden Therapiearme

Bei univariater Betrachtung der Absorption [ml/h] des Pneumozephalus-Volumens zeigen sich Unterschiede im Mittelwert und in der Verteilung der Messwerte in den beiden Therapiegruppen.

7.3.1 Confirmatory *analysis*

Volumendifferenz des Pneumozephalus [ml] CT1 –CT2 Therapie (ANCOVA)
Adjustiert für Baseline des Pneumozephalus-Volumens bei CT1

	100% O2<br>Mean (sdev) (N)	Standard % O2<br>Mean (sdev) (N)	Diff. Means Stand.O2 - 100% O2	95% Confidence Limits Diff. Means		p-value
Gesamt	87.83 (3.31) (22)	71.29 (3.31) (22)	-16.54	-26	-7.08	0.001

Tab.: 6 Volumendifferenz des Pneumozephalus

Absorption des Pneumozephalus-Volumens [ml/h] (CT1 - CT2 / Diff-Time) Therapie (ANCOVA)
Adjustiert für Baseline des Pneumozephalus-Volumens bei CT1.

	100% O2<br>Mean (sdev) (N)	Standard % O2<br>Mean (sdev) (N)	Diff. Means Stand.O2 - 100% O2	95% Confidence Limits Diff. Means		p-value
Gesamt	3.57 (0.13) (22)	2.93 (0.13) (22)	-0.63	-1.01	-0.26	0,0015

Tab.: 7 Absorptionsgeschwindigkeit des Pneumozephalus, adjustiert für das basale Volumen

Die Untersuchung der Volumendifferenz und der Absorptionsgeschwindigkeit für das Volumen des Pneumozephalus nach neurochirurgischer Intervention in halbsitzender Position, zeigt in der Kovarianzanalyse (ANCOVA), mit Adjustierung für das Ausgangsvolumen des Pneumozephalus, signifikante Unterschiede mit einer Überlegenheit der Behandlung mit 100% reinem normobarem Sauerstoff im Vergleich zur Beatmung mit einer Standard-Konzentration von O_2. Somit ist für beide co-primären Endpunkte ein signifikanter Unterschied zu Gunsten der Beatmung mit 100%-O2 festzustellen. Die Studie ist bezüglich ihres primären Studienziels erfolgreich gewesen. Die in der Fallzahlplanung zu Grunde gelegten Unterschiede für die beiden Therapien wurden für die angenommene Volumendifferenz und die Absorptionsrate pro Stunde deutlich überboten.

7.3.2 Sensitivitätsanalyse

Hier folgen die Subgruppen-Analysen für Geschlecht und Alter, wobei die Dichotomisierung des Alters am Median vorgenommen wurde. Ausgewertet wurde die Volumendifferenz des Pneumozephalus [ml] CT1 –CT2 Therapie (ANCOVA) adjustiert für Geschlecht und Baseline des Pneumozephalus-Volumens. bei CT1.

Subgruppen-Analyse für Geschlecht (Diff-Vol.-CT1-CT2)

	100% O2<br>Mean (sdev) (N)	Standard % O2<br>Mean (sdev) (N)	Diff. Means Stand.O2 - 100% O2	95% Confidence Limits Diff. Means		p-value
Gesamt	87.14 (3.21) (22)	71.98 (3.21) (22)	-15.16	-24.34	-5.94	0.002[4]
Male	90.06 (6.01) (8)	74.67 (5.07) (11)	-15.35	-32.49	1.8	0.08
Female	82.11 (3.7) (14)	73.60 (4.22) (11)	-8.50	-20.73	3.74	0.16

Tab.: 8 Volumendifferenz des Pneumozephalus

Es zeigt sich, dass der Effekt der Sauerstofftherapie bei Männern deutlich ausgeprägter als bei Frauen ist. Ein ähnliches Bild zeigt sich auch für die Absorptionsgeschwindigkeit. Aus medizinischer Sicht ist zu prüfen, ob der für Frauen berechnete Therapieeffekt auch relevant ist:

Absorption des Pneumozephalus-Volumens [ml/h] (CT1 - CT2 / Diff-Time) Therapie (ANCOVA)

Adjustiert für Geschlecht und Baseline des Pneumozephalus-Volumens bei CT1.

Subgruppen-Analyse für Geschlecht (Absorption)

	100% O2<br>Mean (sdev) (N)	Standard % O2<br>Mean (sdev) (N)	Diff. Means Stand.O2 - 100% O2	95% Confidence Limits Diff. Means		p-value
Gesamt	3.54 (0.13) (22)	2.95 (0.13) (22)	-0.58	-0.95	-0.22	0.003[4]
Male	3.70 (0.23) (8)	3.10 (0.20) (11)	-0.60	-1.28	0.07	0.08
Female	3.32 (0.15) (14)	2.98 (0.17) (11)	-0.33	-0.83	0.18	0.19

Tab.: 9 Absorptionsgeschwindigkeit des Pneumozephalus

Volumendifferenz des Pneumozephalus [ml] CT1 –CT2 Therapie (ANCOVA)

Adjustiert für Alter und Baseline des Pneumozephalus-Volumens bei CT1
Subgruppen-Analyse für Alter (<= 51.9/>51.9) (Diff-Vol.-CT1-CT2)

	100% O2<br>Mean (sdev) (N)	Standard % O2<br>Mean (sdev) (N)	Diff. Means Stand.O2 - 100% O2	95% Confidence Limits Diff. Means		p-value
Gesamt	87.83 (3.34) (22)	71.16 (3.35) (22)	-16.68	-26.24	-7.11	0.001
Alter: > 51.9 [J]	99.48 (5.63) (9)	74.97 (5.34) (12)	-24.51	-40.79	-8.24	0.005
<= 51.9 [J]	74.61(2.98) (13)	68.59 (3.13) (10)	-6.02	-15.23	3.19	0.19

Tab.: 10 Volumendifferenz des Pneumozephalus

Für die Gruppe der älteren Patienten zeigt sich ein deutlich größerer Therapieeffekt als bei den jüngeren Patienten

Volumendifferenz des Pneumozephalus [ml] CT1 –CT2 Therapie (ANCOVA)
Adjustiert für eine Kombination aus Geschlecht und Alter (dichotom)

	100% O2<br>Mean (sdev) (N)	Standard % O2<br>Mean (sdev) (N)	Diff. Means -Stand.O2 - 100% O2	95% Confidence Limits Diff. Means		p-value
Alter:						
<= 51.9 [J]						
Mann	79.73 (8.05) (3)	76.28 (6.18) (5)	-3.44	-23.91	17.02	0.73
Frau	83.33 (4.88) (8)	75.70 (6.32) (5)	-7.63	-23.99	8.73	0.35
Alter:						
>51.9 [J]						
Mann	86.39 (6.16) (5)	53.47 (6.33) (6)	-32.91	--50.93	-14.90	<0.001
Frau	98.56 (5.64) (6)	81.78 (5.85) (6)	-16.79	-33.51	8.9	-0.049

Tab.: 11 Volumendifferenz des Pneumozephalus

Für die Gruppe der älteren Männer zeigt sich der größte Therapieeffekt.

7.4 Secondary analysis

7.4.1 Stanford Sleepiness Scale

Die sekundäre Analyse umfasst die Messung der Stanford Sleepiness Scale, mit den Werten 1 bis 7, die mit aufsteigendem Wert eine zunehmende Schläfrigkeit beschreibt (Einzelheiten des Test's s. Anlage II)

Post OP. Stanford Sleepiness Scale (SSS) Therapie (ANCOVA)

	100% O2<br>Mean (sdev) (N)	Standard % O2<br>Mean (sdev) (N)	Diff. Means Stand.O2 - 100% O2	95% Confidence Limits Diff. Means		p-value
Gesamt	3.18 (0.3) (22)	4.27 (0.3) (22)	1.1	0.22	1.98	0.015[4]

Tab.: 12 Werte der Stanford Sleepines Scale(Range 1-7), postoperativ im Vergleich der Therapie

Test: [1]CHI2, [2]TTEST, [3]Fisher, [4]Ancova

Bestimmung postoperativ		Therapie<br>100 % O_2	Standard	Gesamt	p-value
					< 0.001[3]
Stanford Sleepiness Scale, kategorial (1-7) N(%) N=44	1	1 (4.5%)	0 (0.0%)	1 (2.3%)	
	2	8 (36.4%)	2 (9.1%)	10 (22.7%)	
	3	6 (27.3%)	6 (27.3%)	12 (27.3%)	
	4	3 (13.6%)	4 (18.2%)	7 (15.9%)	
	5	1 (4.5%)	4 (18.2%)	5 (11.4%)	
	6	3 (13.6%)	6 (27.3%)	9 (20.5%)	

Tab.: 13 Werte der Stanford Sleepines Scale(kategorial), postoperativ im Vergleich der Therapie

Test: [1]CHI2, [2]TTEST, [3]Fischer, [4]Ancova

Die kategoriale Analyse erfolgt hier mit dem exakten Test nach Fisher, da die kategoriale Variable mehr als 2 Ausprägungen hat und teilweise eine geringe Häufigkeit in den einzelnen Zellen ausweist. Die kategoriale und die numerische Analyse zeigen eine deutlich geringere Schläfrigkeit (Score Werte niedriger) für die Gruppe mit der 100%-igen O_2-Therapie. Es wurde keinen Einfluss des Faktors Geschlecht und des Faktors Alter (dichotomisiert) auf den Wert der SSS gefunden.

7.4.2 Farbe-Wort-Interferenz-Test (Stroop)

Zusätzlich werden die Differenzwerte (postoperativ – präoperativ)des Stroop-Tests analysiert (Einzelheiten des Tests s. Anlage I).

- Differenz der Anzahl der Fehler im Wortnamen-Test:

(Post OP – Prä OP). Fehler im Wortnamen-Test Therapie (ANCOVA)

	100% O2 Mean (sdev) (N)	Standard % O2 Mean (sdev) (N)	Diff. Means Stand.O2 - 100% O2	95% Confidence Limits Diff. Means		p-value
Gesamt	0.897 (0.507) (21)	1.272 (0.534) (19)	0.374	-1.1126	1.874	0.62[4]

Tab.: 14 Farbe-Wort-Interferenz (Wortnamen, Anzahl Fehler)im Vergleich der Therapie

Test: [1]CHI2, [2]TTEST, [3]Fisher, [4]Ancova

	100% O2 Anzahl (%) (N)	Standard % O2 Anzahl (%) (N)	p-value
Kein Wortfehler	16 (76.9) (21)	13 (68.4) (19)	0.58[1]

Tab.: 15 Farbe-Wort-Interferenz (Wortnamen), postoperativ im Vergleich der Therapie

Test: [1]CHI2, [2]TTEST, [3]Fisher, [4]Ancova

- Differenz der benötigten Zeit im Wortnamen-Test:
  (Post OP – Prä OP).

	100% O2 Mean (sdev) (N)	Standard % O2 Mean (sdev) (N)	Diff. Means Stand.O2 - 100% O2	95% Confidence Limits Diff. Means		p-value
Gesamt	20.72 (6.81) (21)	26.40 (7.17) (19)	5.67	-14.48	25.83	0.57[4]

Tab.: 16 Farbe-Wort-Interferenz, Differenz der Zeit im Wortnamen-Test

Test: [1]CHI2, [2]TTEST, [3]Fisher, [4]Ancova

Es ergeben sich keine Hinweise auf deutliche Unterschiede für die beiden Therapiegruppen im Wortnamen-Test bezogen auf die Differenz der benötigten Zeit (Post OP – Prä OP) für die Beantwortung und für die Kategorien „Kein Fehler / Mindestens 1 Fehler“

- Differenz der Anzahl der Fehler im Farbnamen-Test:

(Post OP – Prä OP). Fehler im Farbnamen-Test Therapie (ANCOVA)

	100% O2 Mean (sdev) (N)	Standard % O2 Mean (sdev) (N)	Diff. Means Stand.O2 - 100% O2	95% Confidence Limits Diff. Means		p-value
Gesamt	0.97 (0.60) (21)	1.64 (0.64) (18)	0.67	-1.13	2.47	0.45[4]

Tab.: 17 Farbe-Wort-Interferenz (Farbnamen), postoperativ im Vergleich der Therapie

Test: [1]CHI2, [2]TTEST, [3]Fisher, [4]Ancova

	100% O2 Anzahl (%) (N)	Standard % O2 Anzahl (%) (N)	p-value
Kein Farbfehler	10 (66.7) (21)	5 (27.8) (18)	0.20[1]

Tab.: 18 Farbe-Wort-Interferenz (Farbnamen), postoperativ

Test: [1]CHI2, [2]TTEST, [3]Fisher, [4]Ancova

- Differenz der benötigten Zeit im Farbnamen-Test: (Post OP – Prä OP).

	100% O2 Mean (sdev) (N)	Standard % O2 Mean (sdev) (N)	Diff. Means Stand.O2 - 100% O2	95% Confidence Limits Diff. Means		p-value
Gesamt	19.32 (7.89) (21)	25.65 (8.52) (18)	6.32	-17.24	29.89	0.59[4]

Tab.: 19 Farbe-Wort-Interferenz (Farbnamen), Differenz der benötigten Zeit adjustiert für die Baselinezeit im Vergleich der Therapie

Test: [1]CHI2, [2]TTEST, [3]Fisher, [4]Ancova

Die Analyse im Farbnamen- und im Wortnamentest zeigt für die Anzahl der beobachteten Fehler und für die Differenz der für die Beantwortung benötigten Zeit keine deutlichen Unterschiede.

8. Appendix

Anlagen

I. Farbe-Wort-Interferenz-Test
II. Stanford-Sleepines-Scale (SSS)
III. Studienprotokoll
IV. Flowchart der Studie
V. Additive Tabellen
