# Supplementary material for: Normobaric Hyperoxia for Treatment of Pneumocephalus after Posterior Fossa Surgery in the Semisitting Position: A Prospective Randomized Controlled Trial
Source: PLoS One. 2015 May 20;10(5):e0125710. doi: 10.1371/journal.pone.0125710 (PMC4439020; doi:10.1371/journal.pone.0125710)

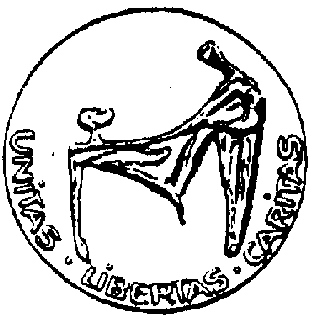


#### **Neurochirurgische Klinik**

##### **Direktor: Prof. Dr. Joachim K. Krauss**

### Carl-Neuberg-Str. 1

30625 Hannover

Telefon: (0511) 532-6652

Fax: (0511) 532-5864

MHH ^⚫^ Neurochirurgie ^⚫^ D-30623 Hannover

Studienprotokoll

Nach Einarbeitung der Vorschläge der Ethikkommission der MHH

und des Instituts für Biometrie der MHH

Version 3.1a

**Prospektive randomisierte Studie zur Analyse der Wirkung von 100% reinem normobarem Sauerstoff zur Behandlung des postoperativen Pneumocephalus nach neurochirurgischen Eingriffen in halbsitzender Position**

____________________ _________________________________________________________

Datum Prof. Dr. Joachim K. Krauss

Projektleitung

____________________ _________________________________________________________

Datum Bujung Hong

Studienarzt

____________________ _________________________________________________________

Datum Dr. Elvis J. Hermann

Studienarzt

____________________ _________________________________________________________

Datum PD Dr. Makoto Nakamura

Studienarzt

____________________ _________________________________________________________

Datum Prof. Dr. Armin Koch

Biometriker

____________________ _________________________________________________________

Datum Anika Großhennig

Biometrikerin

**A. Allgemeine Formalitäten**

**1. Bezeichnung des Vorhabens**

Prospektive randomisierte Studie zur Analyse der Wirkung von 100% reinem normobaren Sauerstoff zur Behandlung des postoperativen Pneumocephalus nach neurochirurgischen Eingriffen in halbsitzender Position.

**2. Projektleitung**

Prof. Dr. med. J. K. Krauss, Direktor der Neurochirurgischen Klinik der MHH, Carl-Neuberg-Str. 1, 30625 Hannover

**3. Angaben über die fachliche Qualifikation des Versuchsleiters gemäß $ 14 des Arzneimittelgesetzes.**

Der Projektleiter ist Facharzt für Neurochirurgie und Direktor der Neurochirurgischen Klinik der MHH. Prof. Krauss verfügt über eine langjährige und umfangreiche operative Erfahrung von pathologischen Prozessen im Bereich der hinteren Schädelgrube sowie des kraniozervikalen Überganges in halbsitzender Position. Hervorzuheben ist insbesondere seine Expertise in der operativen und postoperativen Behandlung von Tumoren und vaskulären Erkrankungen im Kleinhirnbrückenwinkel, Foramen magnum, und im Hirnstammbereich.

Die Anwendung besonderer Pharmaka kommt im Projekt nicht vor.

**4. Namen, Tätigkeiten und Anschriften, der bei der Planung, Durchführung und Auswertung der Studie, beteiligten Ärzte und Wissenschaftler**

Dr. med. E. J. Hermann, Facharzt für Neurochirurgie, Oberarzt der Neurochirurgischen Klinik der MHH, Carl-Neuberg-Str. 1, 30625 Hannover

PD Dr. med. M. Nakamura, Facharzt für Neurochirurgie, Leitender Oberarzt der Neurochirurgischen Klinik der MHH, Carl-Neuberg-Str. 1, 30625 Hannover

B. Hong, wissenschaftlicher Mitarbeiter, Assistenzarzt der Neurochirurgischen Klinik der MHH, Carl-Neuberg-Str. 1, 30625 Hannover

Dr. med. P. Raab, Facharzt für Neuroradiologie, Oberarzt des Institutes für Neuroradiologie der MHH, Carl-Neuberg-Str. 1, 30625 Hannover

PD Dr. med. D. Scheinichen, Facharzt für Anästhesie, Oberarzt der Klinik für Anästhesiologie und Intensivmedizin der MHH, Carl-Neuberg-Str. 1, 30625 Hannover

Prof. Dr. med. J.K. Krauss, Direktor der Neurochirurgischen Klinik der MHH, und PD Dr. med. M. Nakamura, Leitender Oberarzt der Neurochirurgischen Klinik der MHH, haben zahlreiche klinische Studien durchgeführt. Außerdem haben PD Dr. med. M. Nakamura und Dr. med. E. J. Hermann, Oberarzt der Neurochirurgischen Klinik der MHH, eine umfangreiche operative Erfahrung auf dem Gebiet der hinteren Schädelgrube in halbsitzender Position insbesondere unter der Anleitung von Prof. Krauss und haben gemeinsam auch wissenschaftlich dieses Gebiet betreffend bearbeitet. Dr. P. Raab, Oberarzt des Institutes für Neuroradiologie der MHH, verfügt über eine langjährige Erfahrung in der Durchführung und Auswertung unterschiedlichster cerebraler Bildgebungsverfahren, insbesondere der Computertomographie und der Kerspintomographie. PD Dr. med. D. Scheinichen, Oberarzt der Klinik für Anästhesiologie und Intensivmedizin, beherrscht alle fachspezifischen Beatmungstechniken durch seine langjährige Erfahrung in der intensivmedizinischen Behandlung und der Tätigkeit als Leiter der Anästhesie in den neurochirurgischen Operationssälen.

**5. Stellungnahme des ärztlichen verantwortlichen Abteilungsleiters, die sich auf die Sicherstellung der fachlichen, personellen und finanziellen Voraussetzung erstreckt. Mit der Durchführung der wissenschaftlichen Untersuchung einverstanden:**

Prof. Dr. med. J. K. Krauss, Direktor der Neurochirurgischen Klinik der Medizinischen Hochschule Hannover, ist mit der Durchführung der Studie einverstanden.

**6. Finanzierung**

Im Rahmen der Studie erfolgt keine zusätzliche Inanspruchnahme der Krankenkassen oder des Klinikumhaushaltes.

**7. Wurde in der gleichen Sache schon ein Antrag bei einer anderen Ethikkommission gestellt?**

Nein.

**B. Untersuchungsplan:**

**1. Ziel des Vorhabens, Fragestellung und Hypothese**

Eine intrakranielle Luftansammlung, ein sogennanter Pneumocephalus, ist eine der häufigsten Begleiterscheinungen nach den meisten Eingriffen am offenen Schädel (Reasoner et al., 1994). In geringer Form kann ein Pneumocephalus kurzfristig auftreten und wird nach einer bestimmten Zeit reabsorbiert. Obwohl der Pneumocephalus in den meisten Fällen asymptomatisch oder nur leicht symptomatisch ist, kann es jedoch in ausgeprägter Form zu schweren Folgeerscheinungen, wie Bewusstseinsstörung durch massive Kompression des Hirnparenchyms, der Hirnnerven (Markham JW, 1967, Standefer et al., 1984) oder des Hirnstammes (Biyani et al., 2006) führen.

Die halbsitzende Position ist eine Routinelagerung bei Operationen im Bereich der hinteren Schädelgrube. Sie bietet dem Operateur verschiedene Vorteile, wie z.B. eine einfachere anatomische Orientierung und einen relativ blutarmen Operationssitus. Bei bestimmten Operationen bietet auch die natürliche Schwerkraft, die bei der halbsitzenden Position zum Tragen kommt Vorteile (Hermann et al., 2008). Zudem kann die Retraktion des Kleinhirns bei der Operation in halbsitzender Position minimiert werden (Porter et al., 1999). Trotz der gennanten Vorteile kommt es jedoch bei der Operation in halbsitzender Position häufiger zu größeren Luftvolumen durch vermehrten Liquorverlust (Kitahata et al., 1976, MacGilliverg, 1982, Toung et al., 1983, Standefer et al., 1984), die sich meistens frontal finden.

Beim asymptomatischen Pneumocephalus ist eine spontane Resorption, abhängig vom Luftvolumen, zwischen 1 bis 3 Wochen zu erwarten (Di Lorenzo et al., 1986). Im Fall eines symptomatischen postoperativen Pneumocephalus ist eine Bohrlochtrepanation, ggf. mit Anlage einer externen Drainage, häufig erforderlich. Diese zusätzliche Operation ist mit einer zusätzlichen Morbidität und Risiken verbunden.

Über die Gabe von 100%–igem reinen normobaren Sauerstoff zur Behandlung des Pneumocephalus wurde bereits im Rahmen vorläufiger Studien berichtet. Dexter und Reasoner (Dexter et al., 1995) haben durch ein experimentelles mathematisches Model einen Zusammenhang zwischen der Reabsorptionsgeschwindigkeit von N_2_ (Stickstoff) beim Pneumocephalus und den folgenden Faktoren gefunden: der N_2_–Löslichkeit im Blut und Hirngewebe, dem N_2_–Diffusionskoeffizienten im Hirngewebe, der Dichte des Hirngewebes, der Hirndurchblutung, dem partiellen Druck des N_2_ beim Pneumocephalus als auch im Blut. Da die N_2_–Konzentration im umgekehrten Verhältnis zur FiO_2_–Konzentration steht, kann theoretisch eine O_2_–Gabe (Erhöhung der FiO_2_ Konzentration) die Resorption der intrakraniellen Luft beschleunigen. Gore (Gore et al., 2008) zeigte in einer klinischen Fallserie, nämlich bei fünf post–Kraniotomie–Patienten, nach Gabe von 100%igem reinem normobarem Sauerstoff 12L/Minute über eine „Non–Rebreather“ Maske (entspricht FiO_2_ 68%) für 24 Stunden, eine signifikante Reabsorptionsrate der Lufteinschlüsse.

In Anbetracht der Tatsache, dass bei halbsitzender Position ein lebensbedrohlicher Pneumocephalus hervorgerufen werden kann, soll nun, aufbauend auf der Studie von Gore et al., der postoperative Einsatz von 100%–igem reinem normobarem Sauerstoff während der 3-stündigen postoperativen Nachbeatmung über den endotrachealen Tubus analysiert werden.

Das Ziel der Studie ist es, den Nutzen einer Beschleunigung der Reabsorption des postoperativen Pneumocephalus nach Eingriffen im Bereich der hinteren Schädelgrube in halbsitzender Lagerung durch die zusätzliche Therapie mit normobarem 100%–igem Sauerstoff prospektiv randomisiert kontrolliert zu analysieren.

Falls sich eine wesentliche Rolle der reinen normobaren 100% Sauerstoffgabe mit signifikant gesteigerter Resorptionsgeschwindigkeit beim postoperativen Pneumocephalus bestätigen sollte, würde sich neben dem Wissensgewinn für die Pathophysiologie auch ein neuer therapeutischer Ansatz ergeben, welcher schließlich auch zu einer Reduktion der postoperativen Morbidität führen könnte. Um den Zusammenhang zwischen der Sauerstoffgabe und der Reabsorptionsgeschwindigkeit zu untersuchen, planen wir eine dreidimensionelle Rekonstruktion der intrakraniellen Lufteinschlüsse aus den Daten der Computertomographie unmittelbar, spätestens bis 2 Stunden, nach der Operation und nach 24 Stunden nach der Operation zur exakten Beurteilung der Volumenänderung. Als klinische Parameter sollen der Wachheitzustand mittels Stanford Sleepiness Scale (Hoddes et al., 1973) und die eventuelle passagere Funktionsstörung des frontalen Kortex durch die intrakraniellen Lufteinschlüsse mittels Farbe-Wort-Interferenztest (Stroop, 1935; Eigelsreiter et al., 1986) beurteilt werden. Die erhobenen bildgebenden und klinischen Daten werden verglichen und statistisch ausgewertet.

**2. Versuchsaufbau**

Das primäre Ziel dieser prospektiven, kontrollierten randomisierten Studie ist es zu überprüfen, ob die Reabsorption des postoperativen Pneumocephalus nach Eingriffen im Bereich der hinteren Schädelgrube in halbsitzender Lagerung durch die zusätzliche Therapie mit normobarem 100%igem Sauerstoff beschleunigt wird. Hierfür sollen 44 Patienten zwischen 18 und 80 Jahren nach Einwilligung in zwei Gruppen randomisiert werden. Die Therapiegruppe soll postoperativ 100%igen reinen normobaren Sauerstoff über einen endotrachealen Tubus erhalten. Die Kontrollgruppe wird die Standardtherapie erhalten.

Der primäre Endpunkt setzt sich aus zwei co-primären Endpunkten zusammen. Zum einen soll die Veränderung des Luftvolumens im Kopf betrachtet werden. Hierfür wird die Differenz zwischen den Volumina 24 Stunden nach der Extubation und postoperativ mit dem CCT bestimmt. Der zweite co-primäre Endpunkt ist die Absorptionsrate pro Stunde. Diese ergibt sich aus dem Quotienten von Luftvolumenveränderung und postoperativem Volumen multipliziert. Nur wenn die Volumenabnahme und die Absorptionsreduktion in der Therapiegruppe höher sind, ist gezeigt, dass die Sauerstofftherapie der Standardbehandlung überlegen ist.

Ferner soll geklärt werden, ob durch die Therapie die Konzentrationsfähigkeit, gemessen am Farbe-Wort-Interferenztest, der Patienten beeinflusst wird. Darüber hinaus soll die Selbsteinschätzung des Grades der Schläfrigkeit der Patienten (Stanford Sleepiness Scale) zwischen Therapie- und Kontrollgruppe miteinander verglichen werden. Die Erhebung der sekundären Endpunkte erfolgt zeitnah zum CCT.

**3. Randomisierung, Stratifikation, Verblindung**

Die Randomisierung erfolgt nach der Messung der Luftvolumina im Rahmen der ersten postoperativen CT-Kontrolle. Um zu gewährleisten, dass die Therapiezuteilung erst nach Aufnahme in die Studie erfolgt und der Studienarzt folglich keinen Einfluss auf die Therapiezuteilung nehmen kann, wird die Randomisierung zentral durch das Institut für Biometrie der Medizinischen Hochschule Hannover durchgeführt werden. Ferner sollte der Farb-Wort-Interferenztest verblindet gegenüber der Therapie durchgeführt, das bedeutet von einem Untersucher, der keine Kenntnis über die verabreiche Therapie hat.

**4. Fallzahlplannung**

Die primäre Hypothese dieser prospektiven, kontrollierten randomisierten Studie ist, dass die Reabsorption 24 Stunden nach der Extubation bei Patienten mit 3-stundiger 100%igen Sauerstoffgabe über den Tubus nach dem operativen Eingriff, bereits zu einer größeren Volumenveränderung (24h post Extubation – direkt post OP) und einer höheren Absorptionsrate pro Stunde (Volumenänderung / [direkt post OP * Zeit zwischen post-OP- und 24-post-Extubation-Messung]) führt als bei Patienten, die keine 100%ige Sauerstoffgabe über den Tubus erhalten.

Es wird davon ausgegangen, dass es keine Drop-Outs in dieser Studie gibt.

Die Fallzahlplanung basiert auf der Studie von Gore et al. (2008). Die Autoren haben die Volumenveränderung und die Absorptionsrate pro Stunde bei 6 Patienten mit und 7 Patienten ohne Therapie untersucht. Die Volumenveränderung bei den Patienten mit Therapie betrug durchschnittlich 34,6 ml. Bei den Patienten ohne Therapie ergab sich eine durchschnittliche Volumenveränderung von 18 ml. Die gemeinsame Standardabweichung der Volumenveränderung aller Patienten betrug in der Studie von Gore et al. (2008) 16,3 ml. Um bei einer vergleichbaren Variabilität diesen Unterschied zwischen den beiden Gruppenmittelwerten mit einem zweiseitigen t-test für zwei Gruppen bei einer Power von 90% und einem Fehler 1. Art von 5% entdecken zu können sind 22 Patienten pro Gruppe notwendig.

Die Absorptionsrate pro Stunde bei den Patienten mit Therapie betrug durchschnittlich 2,70%. Bei den Patienten ohne Therapie ergab sich eine durchschnittliche Absorptionsrate pro Stunde von 1,33%. Die gemeinsame Standardabweichung der Absorptionsrate pro Stunde aller Patienten betrug in der Studie von Gore et al. (2008) 0,96%. Um bei einer vergleichbaren Variabilität diesen Unterschied zwischen den beiden Gruppenmittelwerten mit einem zweiseitigen t-test für zwei Gruppen bei einer Power von 90% und einem Fehler 1. Art von 5% entdecken zu können sind 12 Patienten pro Gruppe notwendig.

Es wird davon ausgegangen, dass die Auswertung mittels einer ANCOVA die Power erhöht.

Insgesamt sind also mindestens 22 Patienten pro Gruppe notwendig um bei vergleichbaren Patienten, ähnliche Effekte wie in der Studie von Gore et al. (2008) zu entdecken.

**5. Primäre Auswertungsstrategie und Auswertung von wichtigen sekundären Endpunkten**

Die primäre Auswertung erfolgt in der intention-to-treat Population, d.h. alle randomisierten Patienten werden analysiert. Das bedeutet auch für Patienten bei denen nach der Randomisierung festgestellt wird, dass sie ein Einschlusskriterium nicht mehr erfüllen oder ein Ausschlusskriterium erfüllen, wird der primäre Endpunkt erhoben und ausgewertet. Fehlende Werte werden durch die post OP Werte ersetzt. Als Sensitivitätsanalyse wird die Auswertung zusätzlich in der per-protocol Population durchgeführt, d.h. alle Patienten werden entsprechend der verabreichten Therapie ausgewertet.

Für die primäre Analyse wird zunächst für jeden Patienten die Differenz aus den Volumina direkt nach der OP und 24 Stunden nach der Extubation gebildet. Die beiden primären Endpunkte werden jeweils mit einer Kovarianzanalyse (ANCOVA) untersucht. Die abhängige Variable ist der jeweilige Endpunkt. Ferner werden die Behandlung und der Wert des primären Endpunktes direkt nach der Operation in das Modell aufgenommen. Eine größere Volumenänderung unter 100% Sauerstofftherapie wird geschlussfolgert, wenn die untere Grenze des 95% Konfidenzintervalls des Behandlungseffekts größer als 0 ist. Eine höhere Absorptionsrate unter 100% Sauerstofftherapie wird gefolgert, wenn die untere Grenze des 95% Konfidenzintervalls des Behandlungseffekts größer als 0 ist.

Nur wenn für beide Endpunkte eine Überlegenheit der 100% Sauerstofftherapie gezeigt werden kann, ist der Schluss, dass die Therapie dem bisherigen Standard überlegen ist, zulässig.

Alle sekundären Auswertungen sind explorativ. Die sekundären Auswertungen erfolgen analog zur primären Auswertung mittels ANCOVA oder Varianzanalyse (ANOVA). Zur Veranschaulichung werden entsprechend Mittelwerte und Standardabweichungen oder Häufigkeiten angegeben.

Zwischenauswertungen sind nicht geplant.

**6. Einschlusskriterien**

- Alter über 18 Jahre alt

- Standardmäßige Narkose– sowie Beatmungsverhältnisse nach im Haus üblicherweiser verwendeter Methode.

- Regelrechter intraoperative Verlauf ohne Erwartung einer intrakraniellen Komplikation

- Nachweis von intrakraniellen Lufteinschlüsse von mindestens 30 mL Volumen in der

direkten postoperativen CT.

**7. Ausschlusskriterien**

- Patienten mit erwarteter Nachbeatmungszeit.
- Patienten mit intrathorakalen Auffälligkeiten (Z.n. Herz-/Lungeoperation, Tumor, Entzündung, COPD, angeborene Fehlbildung sowie mit schwerer Herzinsuffizienz).
- Patienten mit externer Liquordrainage.
- Schwangere oder stillende Patienten.

**8. Abbruchkriterien**

Die Untersuchung kann auf Wunsch der Patienten jederzeit ohne Angabe von Gründen abgebrochen werden.

**C. Spezielle Formalien**

**1. Art des Vorhabens**

Prospektive, randomisierte klinische Studie ohne Einsatz von Medikamenten.

**2. Finden folgende Bestimmungen Anwendung**

A. Arzneimittelgesetzt : Nein

B. Medizinproduktgesetzt, Medizingeräteverordnung: Nein

C. Gentechnikgesetz: Nein

D. Strahlenschutzverordnung: Nein

E. Röntgenverordnung: Nein

**3. Sind alle für den Menschen ungefährlichen Erprobungsmöglichkeiten ausgeschöpft?**

Es sind keine für den Patienten zusätzliche gefährliche Therapiemaßnahmen oder diagnostische Maßnahmen im Rahmen dieser Studie notwendig. Für die Sauerstoffgabe und Computertomographie gibt es bei Patienten nach Erfüllung der Einschlußkriterien keine Kontraindikationen. Die Computertomographie erfolgt routinemäßig zum Ausschluß einer postoperativen Blutungskomplikation in jedem Falle.

**4. Handelt es sich um eine Untersuchung von Medikamenteneffekten**

Nein.

**5. Abschätzung der wahrscheinlich möglichen Komplikationen und / oder Risiken sowie ihrer Abwägung gegenüber dem voraussichtlichen Nutzen des Vorhabens für den Kranken oder die Allgemeinheit**

Die Studie soll zu einem erweiterten Verständnis der Wirkung von 100% reiner normobarer Sauerstoffgabe bei Auftreten eines postoperativen Pneumocephalus beitragen. Die Sauerstoffgabe über den endotrachealen Tubus im Rahmen unserer Studie sollte für 3 Stunden postoperativ erfolgen. Nebenwirkungen sind bei einer Frist von weniger als 48 Stunden bei streng ausgewählten Patienten praktisch nicht zu erwarten (Jenkinson, 1993). Für die zu untersuchenden Patienten würde eine Minimierung der postoperativen Morbidität durch beschleunigte Luftresorption einen direkten Nutzen bringen. Für die Patienten, die keine 100%ige normobare Sauerstoffgabe erhalten sollten, werden wie üblich, mit der standardisierten Behandlung mit Raumluft über den Tubus nachbeatmet. Hier besteht für die Patienten keinerlei zusätzliche mögliche Komplikation oder Risiko. Ein bestimmter Nutzen ist auch nicht zu erwarten.

**6. Mögliche Komplikationen und Risiken**

Keine

**7. Art der Aufklärung und Einwilligungserklärung der Versuchsperson**

In der Anlage zu diesem Schreiben findet sich unser Entwurf zu einem Aufklärungsbogen und zu einer Einwilligungserklärung für die Studie. Die Teilnahme an der Studie ist freiwillig.

**8. Schweigepflicht und Datenschutz**

Das jeweilige Untersuchungsprotokoll wird in einem Ordner archiviert, der in einem sicher verschlossenen Schrank aufbewahrt wird. Dem Patienten wird eine fortlaufende Nummer im Laborbuch zugeordnet (d.h. Patient 1: 01, Patient 2: 02, usw.), die zur Randomisierung und zur Verschlüsselung der Computerdaten benutzt werden. Die Daten werden separat auf Speichermedien abgespeichert.

# Anlage:

Literaturangaben

Randomisierungsliste

Farbe-Wort-Interferenztest

Standford Sleepiness Scale

Flowchart der Studie

Informationsblatt für Patienten zur Teilnahme an der wissenschaftlichen Untersuchung

Einwilligungserklärung für Patienten zur Teilnahme an einer wissenschaftlichen Untersuchung

# Literaturangaben

Biyani N, Silbiger A, Ben–Ari J, Constantini S. Postoperative brain stem tension pneumocephalus causing transient locked–in syndrome. Pediatr Neurosurg 43:414–417, 2007.

Dexter F, Reasoner DK. Theoretical assessment of nomobaric oxygen therapy to treat pneumocephalus: recommendations for dose and duration of treatment. Anesthesiology 84:442–447, 1996.

Di Lorenzo N, Caruso R, Floris R, Guerrisi V, Bozzao L, Fortuna A. Pneumocephalus and tension pneumocephalus after posterior fossa surgery in the sitting position: a prospective study. Acta Neurochir (Wien) 83:112–115, 1986.

Eigelsreiter H, Ritter M, Weimann J. Untersuchungen mit dem STROOP-Test in großen Höhen. Int Z angew Physiol einschl Arbeitsphysiol 26:13-20, 1986.

Gore PA, Maan H, Chang S, Pitt AM, Spetzler RF, Nakaji P. Normobaric oxygen therapy strategies in the treatment of postcraniotomy pneumocephalus. J Neurosurg 108:926–929, 2008.

Hermann EJ, Rittierodt M, Krauss JK. Combined transventricular and supracerebellar infratentorial approach preserving the vermis in giant pediatric posterior fossa midline tumors. Neurosurgery 63(Suppl1):ONS30–35, 2008.

Hoddes E, Zarcone V, Smythe H, Phillips R, Dement WC. Quantification of sleepiness: a new approach. Psychophysiology 10: 431–436, 1973.

Jenkinson SG. Oxygen toxicity. New Horiz 1:504–511, 1993.

Kitahata LM, Katz JD. Tension pneumocephalus after posterior fossa craniotomy, a complication of the sitting position. Anaesthesiology 44:448–450, 1976.

Mac Gilliverg RG. Pneumocephalus as a complication of posterior fossa surgery in the sitting position. Anesthesia 37:722–725, 1982.

Markham JW. The clinical features of pneumocephalus based upon a survey of 284 cases with report of 11 additional cases. Acta Neurochir(Wien) 16:1–78, 1967.

Porter JM, Pidgeon C, Cunningham AJ. The sitting position in neurosurgery: a critical appraisal. Br J Anaesth 82:117–128, 1999.

Reasoner DK, Todd MM, Scamman FL, Warner DS. The incidence of pneumocephalus after supratentorial craniotomy. Observations on the disappearance of intracranial air. Anesthesiology 80:1008–1012, 1994.

Standefer M, Bay JW, Trusso R. The sitting position in neurosurgery: a retrospective analysis of 488 cases. Neurosurgery 14:649–658, 1984.

Stroop JR. Studies of interference in serial verbal reactions. J Exp Psycho 18: 643–662, 1935.

Toung T, Donham RT, Lehener A, Alano J, Campbell J. Tension pneumocephalus after posterior fossa craniotomy: report of four additional cases and review of postoperative pneumocephalus. Neurosurgery 12:164–168, 1983.


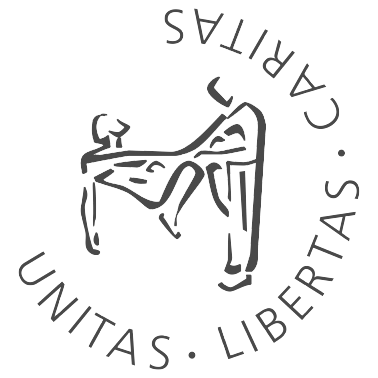


## Farbe-Wort-Interferenztest

( nach J. R. Stroop, 1935 )

**Klinik für Neurochirurgie**

**Direktor : Prof. Dr. med. Joachim K. Krauss**

Bujung Hong

eMail Hong.Bujung@mh-hannover.de

Sekretariat NCH Tel +49–511–532 6651 / 6652

Fax +49–511–532 5864

Carl-Neuberg-Straße 1, 30625 Hannover

Tel +49–511 5320

Web www.mh-hannover.de

Der Farbe-Wort-Interferenztest nach Stroop dient zur Testdiagnostik für exekutive Funktionen des Frontalhirns. Zugrunde liegt der Gedanke, Wortbedeutung und Farbe in einem Stimulus zu kombinieren, und somit eine inkongruente Situation zu schaffen, in welcher sich der semantische Gehalt des Wortes und die Schriftfarbe widersprechen (die Farbe ist niemals identisch mit dem Wortnamen).

1. Aufgabe – Wortnamen

Die Versuchsperson soll so schnell wie möglich die **Wortnamen** laut sagen (die Farbe des Wortes dabei ignorieren, das heißt, das Wort vorlesen). Die Zeit wird mit der Stoppuhr gemessen. Die Fehler werden vermerkt (queres Kreuz über das Farbwort, X).

1. Aufgabe – Farbnamen

Die Versuchsperson soll so schnell wie möglich die Farbe, **Farbnamen**, in der die Wörter geschrieben sind, laut sagen. Die Zeit wird wieder mit der Stoppuhr gemessen. Die Fehler werden wieder vermerkt (queres Kreuz über Farbwort, X)


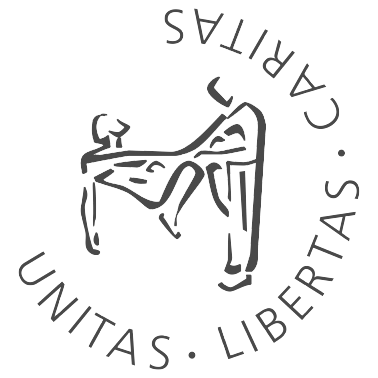


## Farbe-Wort-Interferenztest (Präoperativ)

1. Aufgabe – Wortnamen

## Die Versuchsperson soll so schnell wie möglich von links nach rechts die Wortnamen laut sagen (die Farbe des Wortes dabei ignorieren, das heißt, das Wort vorlesen). Die Zeit wird mit der Stoppuhr gemessen. Die Fehler werden vermerkt (queres Kreuz über Farbwort, X).

**Klinik für Neurochirurgie**

**Direktor : Prof. Dr. med. Joachim K. Krauss**

Bujung Hong

eMail Hong.Bujung@mh-hannover.de

Sekretariat NCH Tel +49–511–532 6651 / 6652

Fax +49–511–532 5864

Carl-Neuberg-Straße 1, 30625 Hannover

Tel +49–511 5320

Web www.mh-hannover.de

Name: _________________________ Datum: __________ ___________

| **Rot** | **Blau** | **Gelb** | **Grün** | **Gelb** | **Grün** | **Gelb** | **Rot** | **Grün** | **Rot** |
| --- | --- | --- | --- | --- | --- | --- | --- | --- | --- |
| **Blau** | **Grün** | **Rot** | **Gelb** | **Rot** | **Grün** | **Grün** | **Blau** | **Rot** | **Gelb** |
| **Grün** | **Blau** | **Rot** | **Blau** | **Blau** | **Rot** | **Grün** | **Blau** | **Grün** | **Blau** |
| **Gelb** | **Grün** | **Rot** | **Grün** | **Gelb** | **Rot** | **Blau** | **Rot** | **Blau** | **Rot** |
| **Rot** | **Gelb** | **Rot** | **Grün** | **Grün** | **Blau** | **Rot** | **Gelb** | **Blau** | **Grün** |

**Zeit: 1. Aufgabe ____________ sec Fehler: 1. Aufgabe ___________**

**Untersucher: _____________________**


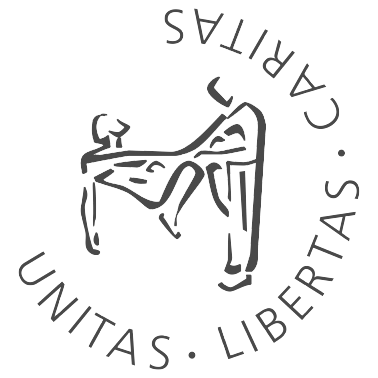


## Farbe-Wort-Interferenztest (Präoperativ)

1. Aufgabe – Farbnamen

Die Versuchsperson soll so schnell wie möglich von links nach rechts die Farbe, **Farbnamen,** in der die Wörter geschrieben sind, laut sagen. Die Zeit wird wieder mit der Stoppuhr gemessen. Die Fehler werden wieder vermerkt (queres Kreuz über Farbwort, X).

**Klinik für Neurochirurgie**

**Direktor : Prof. Dr. med. Joachim K. Krauss**

Bujung Hong

eMail Hong.Bujung@mh-hannover.de

Sekretariat NCH Tel +49–511–532 6651 / 6652

Fax +49–511–532 5864

Carl-Neuberg-Straße 1, 30625 Hannover

Tel +49–511 5320

Web www.mh-hannover.de

Name: _________________________ Datum: __________ ___________

| **Rot** | **Blau** | **Gelb** | **Grün** | **Gelb** | **Grün** | **Gelb** | **Rot** | **Grün** | **Rot** |
| --- | --- | --- | --- | --- | --- | --- | --- | --- | --- |
| **Blau** | **Grün** | **Rot** | **Gelb** | **Rot** | **Grün** | **Grün** | **Blau** | **Rot** | **Gelb** |
| **Grün** | **Blau** | **Rot** | **Blau** | **Blau** | **Rot** | **Grün** | **Blau** | **Grün** | **Blau** |
| **Gelb** | **Grün** | **Rot** | **Grün** | **Gelb** | **Rot** | **Blau** | **Rot** | **Blau** | **Rot** |
| **Rot** | **Gelb** | **Rot** | **Grün** | **Grün** | **Blau** | **Rot** | **Gelb** | **Blau** | **Grün** |

**Zeit: 2. Aufgabe ____________ sec Fehler: 2. Aufgabe ___________**

**Untersucher: _____________________**


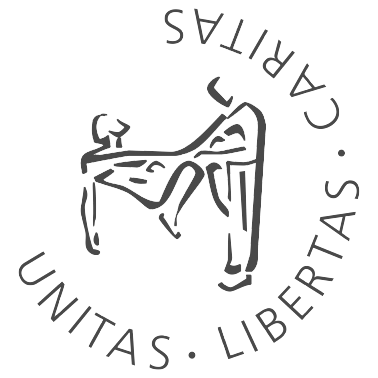


## Farbe-Wort-Interferenztest (Postoperativ)

1. Aufgabe – Wortnamen

## Die Versuchsperson soll so schnell wie möglich von links nach rechts die Wortnamen laut sagen (die Farbe des Wortes dabei ignorieren, das heißt, das Wort vorlesen). Die Zeit wird mit der Stoppuhr gemessen. Die Fehler werden vermerkt (queres Kreuz über Farbwort, X).

**Klinik für Neurochirurgie**

**Direktor : Prof. Dr. med. Joachim K. Krauss**

Bujung Hong

eMail Hong.Bujung@mh-hannover.de

Sekretariat NCH Tel +49–511–532 6651 / 6652

Fax +49–511–532 5864

Carl-Neuberg-Straße 1, 30625 Hannover

Tel +49–511 5320

Web www.mh-hannover.de

Name: _________________________ Datum: __________ ___________

| **Rot** | **Blau** | **Gelb** | **Grün** | **Gelb** | **Grün** | **Gelb** | **Rot** | **Grün** | **Rot** |
| --- | --- | --- | --- | --- | --- | --- | --- | --- | --- |
| **Blau** | **Grün** | **Rot** | **Gelb** | **Rot** | **Grün** | **Grün** | **Blau** | **Rot** | **Gelb** |
| **Grün** | **Blau** | **Rot** | **Blau** | **Blau** | **Rot** | **Grün** | **Blau** | **Grün** | **Blau** |
| **Gelb** | **Grün** | **Rot** | **Grün** | **Gelb** | **Rot** | **Blau** | **Rot** | **Blau** | **Rot** |
| **Rot** | **Gelb** | **Rot** | **Grün** | **Grün** | **Blau** | **Rot** | **Gelb** | **Blau** | **Grün** |

**Zeit: 1. Aufgabe ____________ sec Fehler: 1. Aufgabe ___________**

**Untersucher: _____________________**


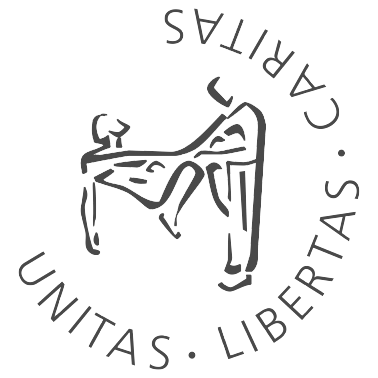


## Farbe-Wort-Interferenztest (Postoperativ)

1. Aufgabe – Farbnamen

Die Versuchsperson soll so schnell wie möglich von links nach rechts die Farbe, **Farbnamen**, in der die Wörter geschrieben sind, laut sagen. Die Zeit wird wieder mit der Stoppuhr gemessen. Die Fehler werden wieder vermerkt (queres Kreuz über Farbwort, X)

**Klinik für Neurochirurgie**

**Direktor : Prof. Dr. med. Joachim K. Krauss**

Bujung Hong

eMail Hong.Bujung@mh-hannover.de

Sekretariat NCH Tel +49–511–532 6651 / 6652

Fax +49–511–532 5864

Carl-Neuberg-Straße 1, 30625 Hannover

Tel +49–511 5320

Web www.mh-hannover.de

Name: _________________________ Datum: __________ ___________

| **Rot** | **Blau** | **Gelb** | **Grün** | **Gelb** | **Grün** | **Gelb** | **Rot** | **Grün** | **Rot** |
| --- | --- | --- | --- | --- | --- | --- | --- | --- | --- |
| **Blau** | **Grün** | **Rot** | **Gelb** | **Rot** | **Grün** | **Grün** | **Blau** | **Rot** | **Gelb** |
| **Grün** | **Blau** | **Rot** | **Blau** | **Blau** | **Rot** | **Grün** | **Blau** | **Grün** | **Blau** |
| **Gelb** | **Grün** | **Rot** | **Grün** | **Gelb** | **Rot** | **Blau** | **Rot** | **Blau** | **Rot** |
| **Rot** | **Gelb** | **Rot** | **Grün** | **Grün** | **Blau** | **Rot** | **Gelb** | **Blau** | **Grün** |

**Zeit: 1. Aufgabe ____________ sec Fehler: 1. Aufgabe ___________**

**Untersucher: _____________________**


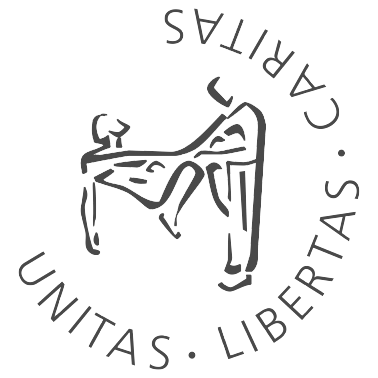


**Klinik für Neurochirurgie**

**Direktor : Prof. Dr. med. Joachim K. Krauss**

Bujung Hong

eMail Hong.Bujung@mh-hannover.de

Sekretariat NCH Tel +49–511–532 6651 / 6652

Fax +49–511–532 5864

Carl-Neuberg-Straße 1, 30625 Hannover

Tel +49–511 5320

Web www.mh-hannover.de

**Stanford Sleepiness Scale**

( Hoddes et al., 1973 )

MHH Neurochirurgie 30625 Hannover

Der Patient wird gebeten, seine Vigilanz auf dieser Skala einzuordnen. Der Wert entspricht dem höchstmöglichen Grad der Wachheit, während ein Wert von 7 den Grad größtmöglicher Schläfrigkeit darstellt.

**Name: _________________________**  Datum: __________ ___________

| **Grad der Schläfrigkeit** | **Scales**  **Rating** |
| --- | --- |
| Fühle mich aktiv, vital, voll da, hellwach | 1 |
| Habe einen klaren Kopf, bin aber nicht in Top-Form: kann mich konzentrieren | 2 |
| Wach, aber entspannt; reagiere, bin aber nicht so ganz da | 3 |
| Etwas benommen, schlaff | 4 |
| Benommen, verliere das Interesse am Wachbleiben, tranig | 5 |
| Schläfrig, benommen, kämpfe mit dem Schlaf; würde mich gerne hinlegen | 6 |
| Kämpfe nicht mehr gegen den Schlaf, schlafe gleich ein; traumartige Gedanken | 7 |
| Schlafe | X |

**Untersucher : ______________________**


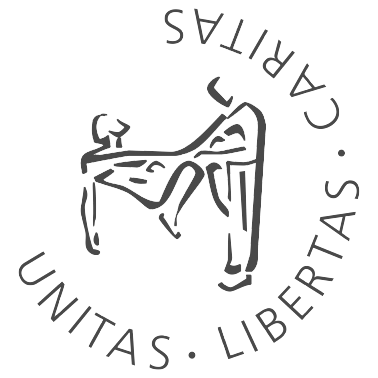


**Klinik für Neurochirurgie**

**Direktor : Prof. Dr. med. Joachim K. Krauss**

Bujung Hong

eMail Hong.Bujung@mh-hannover.de

Sekretariat NCH Tel +49–511–532 6651 / 6652

Fax +49–511–532 5864

Carl-Neuberg-Straße 1, 30625 Hannover

Tel +49–511 5320

Web www.mh-hannover.de

**Flowchart der Studie**

**Titel der Studie :**

MHH Neurochirurgie 30625 Hannover

Prospektive randomisierte Studie zur Analyse der Wirkung von 100% reinem normobaren Sauerstoff zur Behandlung des postoperativen Pneumocephalus

nach neurochirurgischen Eingriffen in halbsitzender Position.

**Patientenselektion, Aufklärung, Farbe-Wort-Interferenztest**

**Operation, Wundverschluß**

**BGA Kontrolle**

**< 2 Stunden**

**CCT mit Rekonstruktion**

**Intrakranielle Luft > 30 mL ?**

**Ja Nein keine Teilnahme**

**an der Studie**

**Randomisierung**

**Gruppe 1 Gruppe 2**

**BIPAP Beatmung Übliches Procedere**

**100% O_2_ über den Tubus (Extubation, spontane Atmung,**

**BGA Kontrolle)**

**3 Stunden**

**Extubation, BGA Kontrolle**

**Falls noch nicht extubabel:**

**weitere O_2_ über den Tubus**

**nach Bedarf Erhebung primärer Endpunkt:**

**CCT 24 Stunden nach der O_2_ Gabe**

**bzw. nach der stationären Aufnahme**

**Erhebung sekundärer Endpunkte:**

**Stanford Sleepiness Scale,**

**Farbe-Wort-Interferenztest zeitnah zum CCT**

**Informationsblatt für Patienten**

**zur Teilnahme an der wissenschaftlichen Untersuchung**

**Klinik für Neurochirurgie**

**Direktor : Prof. Dr. med. Joachim K. Krauss**

Bujung Hong

eMail Hong.Bujung@mh-hannover.de

Sekretariat NCH Tel +49–511–532 6651 / 6652

Fax +49–511–532 5864

Carl-Neuberg-Straße 1, 30625 Hannover

Tel +49–511 5320

Web www.mh-hannover.de

**Titel der Studie :**

Prospektive randomisierte Studie zur Analyse der Wirkung von 100% reinem normobaren Sauerstoff zur Behandlung des postoperativen Pneumocephalus nach neurochirurgischen Eingriffen in halbsitzender Position.

Sehr geehrte Patientin, sehr geehrter Patient,

in der Medizinischen Hochschule Hannover arbeiten wir ständig daran, die Versorgung unserer Patienten zu verbessern. Zu diesem Zweck gehört auch die Durchführung von klinischen Studien und Nachuntersuchungen. Aktuell führen wir eine Studie über die Gabe von 100%igem reinem Sauerstoff bei normalem Druck zur Behandlung postoperativer Luftannsammlungen innerhalb des Schädels nach einer Operation in halbsitzender Position durch.

Aufgrund Ihrer Krankheit ist bei Ihnen eine neurochirurgische Operation erforderlich und diese soll in halbsitzender Position durchgeführt werden. Dabei kommt es häufig zur vermehrten Luftansammlungen innerhalb des Schädels durch vermehrten Verlust des Hirnwassers während der Operation.

In den meisten Fällen ist dies ohne Krankheitszeichen. Selten kann es aber durch eine raumfordernde Wirkung der Luft auch zu Kopfschmerzen, im Extremfall zu einer Bewußtseinsstörung kommen. Studien in der Vergangenheit geben Hinweise darauf, dass die Gabe von 100% reinem Sauerstoff bei normalem Druck nach der Operation die Abnahme der Luft im Schädel beschleunigen kann. Ob dies tatsächlich der Fall ist, muß in einer zufallszugeteilten Studie allerdings noch bestätigt werden. Durch unsere Studie soll die Abnahme der Luftansammlungen im Schädel bei Operationen in halbsitzender Position durch die Wirkung der Sauerstoffgabe erfasst werden. Das Ergebnis der Studie wird zur weiteren Bearbeitung und Optimierung der postoperativen Behandlungsmethode angewendet werden.

Bei allen Patienten wird zunächst ein Farbe-Wort-Interferenztest nach Stroop vor der Operation durchgeführt. Mit diesem Test kann man die Konzentrationsfähigkeit untersuchen. Unmittelbar nach der Operation wird routinemäßig eine Computertomographie des Schädels durchgeführt. Falls das Volumen der Luftansammlung über 30 mL ist, werden Sie in die Studiengruppe aufgenommen. Falls Sie nach einer zufallsgesteurten Verteilung (1:1 mit einem Computerprogramm) der Behandlungsgruppe zugeteilt würden, wird während der Nachbeatmungszeit die Gabe von 100% reinem Sauerstoff über den Beatmungsschlauch erfolgen. Anschließend soll eine weitere Computertomographie des Schädels sowie ein zweiter Farbe-Wort-Interferenztest nach Stroop und eine Wachheitsbeurteilung mittels der Stanford Sleepiness Scale nach 24 Stunden erfolgen. Ein Risiko für Sie ist bei kurzfristiger Sauerstoffsgabe im Rahmen unserer Studie nicht gegeben.

###### Information zum Datenschutz

Ihre im Rahmen dieser klinischen Studie erhobenen personenbezogenen Daten und Gesundheits- bzw. Krankheitsdaten, sowie die Ergebnisse der im Rahmen der Versuchsteilnahme erfolgten Untersuchungen und Behandlung werden im Falle Ihrer Studienteilnahme zu diesem Zweck wie folgt verarbeitet:

Ihre persönlichen Daten: Name, Vorname, Geschlecht und Geburtsdatum und Adresse werden durch den Sie aufklärenden Prüfarzt erhoben und von diesem auf der Einwilligungserklärung vermerkt. Die Einwilligungserklärung mit diesen unverschlüsselten personenbezogenen Angaben bleibt bei dem/der Studienarzt / Studienärztin. Die im Rahmen dieser Studie erhobenen und gewonnenen Gesundheits- bzw. - Krankheitsdaten und die Ergebnisse der klinischen Studie werden anonymisiert und in dieser Form elektronisch gespeichert. Der Schlüssel, der gebraucht wird, um diese Daten Ihren persönlichen Daten zuordnen zu können, ist auf einem von den übrigen Daten getrennten Computer bei dem / der Studienarzt / Studienärztin gespeichert und nur für diesen zugänglich.

Die Ergebnisse der Studie werden ohne Nennung Ihres Namens oder anderer Ihre Person erkennenlassender Angaben (d.h. anonym) veröffentlicht.

Ihre gespeicherten oder sonst aufgezeichneten Daten/personenbezogenen Angaben und der Schlüssel zu ihrer Entschlüsselung werden bis zur Veröffentlichung der Studie gespeichert bzw. archiviert und danach gelöscht bzw. vernichtet. Die ggf. in Ihrer Krankenakte befindlichen Aufzeichnungen, welche in Bezug zu der o.g. Studie stehen, werden 30 Jahre aufbewahrt und danach mit dieser vernichtet.

Sie können jederzeit einer Weiterverarbeitung Ihrer Daten widersprechen. In diesem Fall werden die über Sie gespeicherten persönlichen Angaben und der dazugehörende Schlüssel gelöscht bzw. vernichtet. Ferner können Sie unrichtige Daten, die Sie betreffen, berichtigen lassen.

Fragen zu allen Angelegenheiten, welche die Studie betreffen, insbesondere auch über Risiken können jederzeit an folgende Studienärzte gerichtet werden:

PD Dr. med. M. Nakamura, Neurochirurgische Klinik, Tel. 0511–532 3770.

Dr. med. E. J. Hermann, Neurochirurgische Klinik, Tel. 0511–532 2359.

B. Hong, Neurochirurgische Klinik, Tel. 0511–532 3101.

Die Teilnahme an der Studie erfolgt freiwillig. Sie können jederzeit von der wissenschaftlichen Untersuchung ohne Angabe von Gründen zurücktreten, ohne dass hierdurch die ansonsten bei Ihnen notwendigen medizinischen Maßnahmen beeinträchtigt werden. Gleiches gilt für den Abbruch der Studienteilnahme und den Widerruf der Einwilligung sowie bei einem Widerspruch gegen die Weiterverarbeitung der Daten.


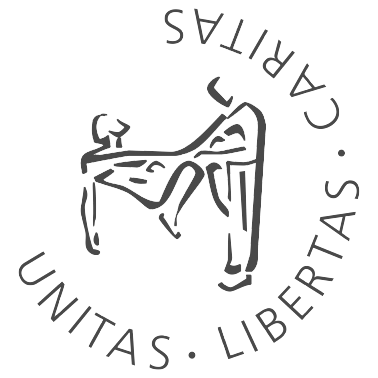


**Einwilligungserklärung für Patienten zur Teilnahme an der wissenschaftlichen Untersuchung**

**Titel der Studie :**

Prospektive randomisierte Studie zur Analyse der Wirkung von 100% reinem normobaren Sauerstoff zur Behandlung des postoperativen Pneumocephalus

nach neurochirurgischen Eingriffen in halbsitzender Position.

Ich bin von Dr. ... ... ... ... ... ... über folgenden Sachverhalt aufgeklärt worden :

**Klinik für Neurochirurgie**

**Direktor : Prof. Dr. med. Joachim K. Krauss**

Bujung Hong

eMail Hong.Bujung@mh-hannover.de

Sekretariat NCH Tel +49–511–532 6651 / 6652

Fax +49–511–532 5864

Carl-Neuberg-Straße 1, 30625 Hannover

Tel +49–511 5320

Web www.mh-hannover.de

durch die Studie soll die Wirkung der Gabe von 100% reinem Normaldrucksauerstoff zur Behandlung postoperativer Lufteinschlüße in der Schädelhöhle nach Operation in halbsitzender Position untersucht werden.

Ich habe die Ziele und die Durchführung der Untersuchung verstanden. Ich habe die schriftliche Information zur Durchführung der Untersuchung gelesen, bin zusätzlich mündlich von dem Studienarzt / der Studienärztin über die Untersuchung aufgeklärt worden und habe keine weiteren unbeantworteten Fragen. Ich unterziehe mich der Untersuchung freiwillig und weis, dass ich diese jederzeit ohne Nachteile für die Behandlung abbrechen kann.

Ich bin mit der Aufzeichnung meiner Krankheitsdaten und der Erhebung von Untersuchungsbefunden einverstanden. Alle personenbezogenen Daten unterliegen der Schweigepflicht und dem Bundesdatenschutzgesetz.

### Hannover, den

______________________________________ _______________________________________

( Arzt ) ( Patient )


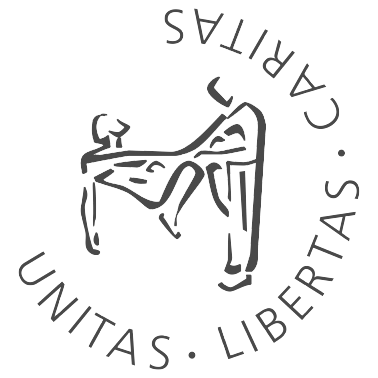

Supplement: S1 Protocol — (DOC) [file pone.0125710.s004.doc]
