# Supplementary material for: Normobaric Hyperoxia for Treatment of Pneumocephalus after Posterior Fossa Surgery in the Semisitting Position: A Prospective Randomized Controlled Trial
Source: PLoS One. 2015 May 20;10(5):e0125710. doi: 10.1371/journal.pone.0125710 (PMC4439020; doi:10.1371/journal.pone.0125710)
Supplement: S2 Protocol — (DOC) [file pone.0125710.s005.doc]

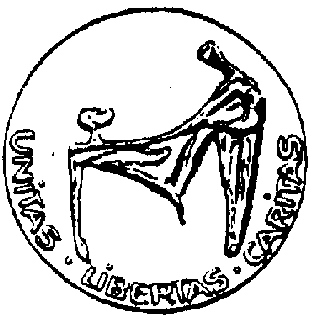


#### **Neurochirurgische Klinik**

##### **Direktor: Prof. Dr. Joachim K. Krauss**

### Carl-Neuberg-Str. 1

30625 Hannover

Telefon: (0511) 532-6652

Fax: (0511) 532-5864

MHH ^⚫^ Neurochirurgie ^⚫^ D-30623 Hannover

**Clinical study protocol**

**Normobaric hyperoxia for treatment of pneumocephalus after posterior fossa surgery in the semisitting position:**

**a prospective randomized controlled trial**

Following incorporation of the Ethic Committee of Hannover Medical School and Institute for Biostatistics of Hannover Medical School advisers` proposal

Protocol version : 3.1.a

Protocol Date : 19 October 2010

Project Leader : Joachim K. Krauss, M.D.

Protocol Author(s) : Bujung Hong, M.D., Elvis J. Hermann, M.D., Anika Grosshennig, PhD.,

Armin Koch, M.D., Makoto Nakamura, M.D., Joachim K. Krauss, M.D.

____________________ _________________________________________________________

Date Prof. Dr. Joachim K. Krauss

Project Leader

____________________ _________________________________________________________

Date Bujung Hong

Investigator

____________________ _________________________________________________________

Date Dr. Elvis J. Hermann

Investigator

____________________ _________________________________________________________

Date PD Dr. Makoto Nakamura

Investigator

____________________ _________________________________________________________

Date Prof. Dr. Armin Koch

Biometrician

____________________ _________________________________________________________

Date Anika Grosshennig

Biometrician

1. **General formalities**
2. **Title**

Normobaric hyperoxia for treatment of pneumocephalus after posterior fossa surgery in the semisitting position: a prospective randomized controlled trial

1. **Project Leader**

Prof. Dr. med. Joachim K. Krauss, Director and Chairman, Department of Neurosurgery, Hannover Medical School, Carl-Neuberg-Strasse 1, 30625 Hannover, Germany

1. **Qualifications of the project leader in accordance to § 14 of German Medicine Act**

Prof. Dr. med. J.K. Krauss is Director and Chairman of the Department of Neurosurgery of Hannover Medical School. Professor Krauss has a long-standing and extensive experience in the surgical resection of various lesions in posterior fossa and craniocervical junction in semisitting position, particularly tumors and vascular disorders in cerebellopontine angle, foramen magnum, and brainstem.

1. **Project Investigators**

Dr. med. E. J. Hermann, Consultant Neurosurgeon, Department of Neurosurgery, Hannover Medical School, Carl-Neuberg-Str. 1, 30625 Hannover

PD Dr. med. M. Nakamura, Consultant Neurosurgeon, Department of Neurosurgery, Hannover Medical School, Carl-Neuberg-Str. 1, 30625 Hannover

B. Hong, Resident, Department of Neurosurgery, Hannover Medical School, Carl-Neuberg-Str. 1, 30625 Hannover

Dr. med. P. Raab, Consultant Neuroradiologist, Institute of Diagnostic and Interventional Neuroradiology, Hannover Medical School, Carl-Neuberg-Str. 1, 30625 Hannover

PD Dr. med. D. Scheinichen, Consultant Anaesthesist, Department of Anaesthesiology Hannover Medical School, Carl-Neuberg-Str. 1, 30625 Hannover

Prof. Dr. med. J.K. Krauss, Director and Chairman, Department of Neurosurgery, Hannover Medical School, Carl-Neuberg-Str. 1, 30625 Hannover

**5. Statement of medical director/chairman of the department on responsibility for the conduct of the study:**

Prof. Dr. med. J. K. Krauss, Director and Chairman of Department of Neurosurgery Hannover Medical School, agrees with the trial.

1. **Funding**

No additional costs of health insurance or hospital budget are required for this study.

**7. Were there any applications for ethical approval concerning the same issue?**

No.

1. **Study Procedures:**
2. **Aim of study, objective, hypothesis**

Accumulation of intracranial air is one of accompanying appearances following cranial surgeries (Reasoner et al., 1994).Small amount of intracranial air is reabsorbed completely within days or weeks, and in most instances, no clinical sign or symptom was observed. Accumulation of large amounts of intracranial air is rare, however, it can develop into a serious emergency situation, particularly if air pressure increases, so-called tension pneumocephalus, and compresses the brain and the cranial nerves extensively (Markham JW, 1967; Standefer et al., 1984), and even life-threatening brainstem compression or brain herniation (Biyani et al., 2006).

Surgical interventions in the semisitting position are intended to optimize surgical conditions by reducing bleeding in the operation field, reducing brain swelling, providing a clean view of the surgical field, and improving the surgical approach. The gravitational effects contribute the better anatomical orientation and less brain retraction (Hermann et al., 2008; Porter et al., 1999). There are, however, some potentially life-threatening risks associated with surgery in the semisitting position, among others, pneumocephalus (Kitahata et al., 1976, MacGilliverg, 1982, Toung et al., 1983, Standefer et al., 1984).

Asymptomatic pneumocephalus would be reabsorbed spontaneously within 1 to 3 weeks (Di Lorenzo et al., 1986). Large symptomatic intracranial air might require emergency twist-drill or burr hole craniostomy to remove the entrapped air. This procedure is associated with additional morbidity and surgical risks.

Normobaric oxygen has been suggested to be effective to treat pneumocephalus, since oxygen is able to replace nitrogen by increasing the diffusion gradient for nitrogen between the air collection and the surrounding tissue (Dexter & Reasoner, 1996). Using a mathematical model, proposed by Dexter and Reasoner in 1996, a FiO_2_ of 100% would be necessary to achieve significant air absorption within hours to days (Dexter & Reasoner, 1996). This model suggests that air would be resorbed faster if there is a greater gradient in the concentration of molecules between air and blood. The air resorption rate would be higher if there is less N_2_ dissolved in the blood of capillary arteries in the adjacent cerebral tissue. In 2008, Gore et al. demonstrated a significant increase of pneumocephalus resorption in 6 patients, who had normobaric hyperoxia at FiO_2_ 68% over a non-rebreather mask for a period of 24 hours, resulting in a significant mean air volume reduction (Gore et al., 2008).

Considering that, we aimed to analyze the effect of administration of normobaric hyperoxia at FiO_2_ 100% over an endotracheal tube (ETT) for 3 hours on pneumocephalus after posterior fossa surgery in the semisitting position. If higher air resorption rate were to be confirmed, a novel therapy approach will be established to reduce the postoperative morbidity.

Routine cranial CT scans with a secondary reconstruction of the volume of intracranial air will be performed immediately and 24 hours after surgery.

To assess the clinical state, patients will be requested to indicate their own level of sleepiness according to the Stanford Sleepiness Scale (Hoddes et al., 1973). Moreover, possible temporary frontal lobe dysfunction which correlates to pneumocephalus, will be assessed using the Stroop color-word interference test (Stroop JR, 1935; Eigelsreiter et al., 1986). The collected data and images will be compared and analysed.

1. **Study objectives**

The aim of this prospective randomized controlled trial is to prove, whether administration of normobaric hyperoxia at FiO_2_ 100% might accelerate the resorption of postoperative supratentorial pneumocephalus after posterior fossa surgery in the semisitting position.

A total of 44 patients aged 18 to 80 years will be randomized and evaluated after written informed consent are obtained. Patients randomized to the treatment group will be ventilated with normobaric hyperoxia at FiO_2_ 100% via the endotracheal tube (ETT). Patients randomized to the control group will be weaned off from ventilation and will then be extubated.

The primary objective of this study consists of two co-primary endpoints. The first co-primary endpoint is the mean change of air volume between CT scan performed immediately and 24 hours after completion of surgery. The second co-primary endpoint is the air resorption rate, which is defined as the mean change of air volume divided by the time between the two CT scans.

Furthermore, the efficacy of normobaric hyperoxia at FiO_2_ of 100% on attention, as measured by the Stroop color-word interference tests, and on alertness, as noted on the Stanford Sleepiness Scale, will be compared and analyzed.

**3. Randomization procedure**

Patients meeting the inclusion criteria will be randomized to treatment or control group after measurement of the air volume in cranial CT scan which will be performed immediately after completion of surgery. The randomization will be conducted independently from the patient recruitment at the Institute of Biostatistics. The Stroop color-word interference test will be performed one day before surgery and 24 hours after surgery by an Investigator, which is blinded to the treatment group.

1. **Patients selection**

We hypothesized that patients who will be treated with normobaric hyperoxia at FiO_2_ 100% via endotracheal tube for 3 hours will show a significantly volume change and a higher resorption rate of postoperative pneumocephalus as compared to untreated patients. It is assumed, that there are no drop-outs in this trial.

Sample size is calculated based on the study by Gore et al (2008). In this study, 6 patients, who had normobaric hyperoxia at FiO_2_ 68%, over a non-rebreather mask for a period of 24 hours, were compared to 7 patients who were breathing room air. Assuming a two-sided type I error of 5% and a power of 90%, for the mean change of pneumocephalus a total of 22 patients per study group (difference:16.6, common SD: 16.3) and for the mean air absorption rate (difference:1.37, common SD: 0.96) a total of 12 patients per study group are considered necessary. Subsequently, a total of 44 patients are necessary for recruitment. Moreover, it is assumed that the ANCOVA may enhance the statistic power.

**5. Statistical consideration**

Baseline characteristics (quantitative parameters: mean, standard deviation (SD), range; qualitative parameters: absolute and relative frequencies) will be compared descriptively using two-sided t-tests or χ^2^ tests.

Both primary endpoints will be analysed using an analysis of covariance (ANCOVA) including the respective baseline values. A 95% confidence interval for the treatment effect (control – treatment) and the corresponding p-values will be calculated from the ANCOVA model. A two-sided p-value less than 0.05 is considered statistically significant. Estimates of the mean change and respective standard errors (SE) from the ANCOVA model per study group will be calculated. Sensitivity analysis including gender and age (dichotomized by the respective median) and the respective subgroups will be reported separately.

Differences in mean time for the Stroop color-word interference tests (postoperative – preoperative) will be analyzed in line with the primary endpoints. In addition no failure versus one or more failures in word reading/colour naming will be compared between the treatment and control group using descriptive χ^2^ tests.

The Stanford Sleepiness Scale will be analysed using descriptive two-sided t-test and Fisher’s exact test.

1. **Inclusion criteria**

Primary inclusion criteria are patient’s age above 18 years, who will undergo posterior fossa surgery in the semi-sitting position (as performed routinely in ours institution). Surgery must be performed with standard anaesthetic and ventilation procedures. Postoperative course must be uneventful without any complication. CT scan should show intracranial air collection of 30 ml or more.

1. **Exclusion criteria**

Patients with a history of any cardiac disease or previously cardiac surgery, any pulmonary disease, chronic obstructive pulmonary disease (COPD), chronic cough or dyspnoe, abnormal chest X-ray, or previously pulmonary surgery are not considered for this study.

Patients, who will need, for whatever reason, a CSF drain or prolonged postoperative ventilation, will be excluded.

Pregnant or breastfeeding women will also be excluded.

1. **Discontinue criteria**

Patients may discontinue from participation in this study at any time without giving any reasons.

1. **Special formalities**

**1. Study design**

A prospective, randomized, observer-blind, controlled clinical study is planned to involve 44 adult patients with pneumocephalus after posterior fossa surgery in the semisitting position.

**2. Application for the following regulations:**

A. Medicine Act: No

B. Medical Products Law, Medical Devices Act: No

C. Genetic Engineering Law: No

D. Radiation Protection Regulation: No

E. X-Ray Regulation: No

**3. Have all non-hazardous trial possibilities carefully been considered?**

For this study are no additional and potential dangerous treatment and/or diagnostic measurements necessary. There are no contraindications for normobaric hyperoxia at FiO_2_ 100% and/or postoperative cranial CT scan for patients that have been included in the study. A postoperative cranial CT scan will be routinely performed irrespectively to exclude complications like brain hemorrhage or infarction.

**4. Is this an investigation concerning effectiveness of drug therapy?**

No, there is no medication involved in this study.

1. **Patient safety and consideration of benefits**

This study should extend the understanding on the effect of normobaric hyperoxia at FiO_2_ 100% on postoperative pneumocephalus. Patients in the treatment group will be ventilated with FiO_2_ 100% over the endotracheal tube for a period of 3 hours. No adverse effects are expected after administration of normobaric hyperoxia in time duration of less than 48 hours on strictly selected patients (Jenkinson, 1993).

For the patients in the treatment group, acceleration of the air resorption of pneumocephalus might minimize the postoperative morbidity. Patients in the control group will be ventilated with room air until extubation according to standardized treatment. Thus, no additional complications or risks are expected. A specific benefit is also not expected.

**6. Possible complications and risks**

None.

**7. Patient information and consent**

Patient Information Sheet and Informed Consent Form are attached. Patients will be notified that they are free to participate and also free to discontinue from the study at any time.

**8. Patient data protection**

The Study Protocols and Informed Consent Form will be stored in a securely locked cabinet, maintaining confidentiality. Patients will be identified by subject number only for the purpose of randomization and encoding the computer database.

# Attachment:

Bibliographic references

Stroop color-word interference test

Standford Sleepiness Scale

Study flow charts

Patients Information Sheet

Informed Consent Form

**BIBLIOGRAPHIC REFERENCES**

Biyani N, Silbiger A, Ben–Ari J, Constantini S. Postoperative brain stem tension pneumocephalus causing transient locked–in syndrome. Pediatr Neurosurg 43:414–417, 2007.

Dexter F, Reasoner DK. Theoretical assessment of nomobaric oxygen therapy to treat pneumocephalus: recommendations for dose and duration of treatment. Anesthesiology 84:442–447, 1996.

Di Lorenzo N, Caruso R, Floris R, Guerrisi V, Bozzao L, Fortuna A. Pneumocephalus and tension pneumocephalus after posterior fossa surgery in the sitting position: a prospective study. Acta Neurochir (Wien) 83:112–115, 1986.

Eigelsreiter H, Ritter M, Weimann J. Untersuchungen mit dem STROOP-Test in großen Höhen. Int Z angew Physiol einschl Arbeitsphysiol 26:13-20, 1986.

Gore PA, Maan H, Chang S, Pitt AM, Spetzler RF, Nakaji P. Normobaric oxygen therapy strategies in the treatment of postcraniotomy pneumocephalus. J Neurosurg 108:926–929, 2008.

Hermann EJ, Rittierodt M, Krauss JK. Combined transventricular and supracerebellar infratentorial approach preserving the vermis in giant pediatric posterior fossa midline tumors. Neurosurgery 63(Suppl1):ONS30–35, 2008.

Hoddes E, Zarcone V, Smythe H, Phillips R, Dement WC. Quantification of sleepiness: a new approach. Psychophysiology 10: 431–436, 1973.

Jenkinson SG. Oxygen toxicity. New Horiz 1:504–511, 1993.

Kitahata LM, Katz JD. Tension pneumocephalus after posterior fossa craniotomy, a complication of the sitting position. Anaesthesiology 44:448–450, 1976.

Mac Gilliverg RG. Pneumocephalus as a complication of posterior fossa surgery in the sitting position. Anesthesia 37:722–725, 1982.

Markham JW. The clinical features of pneumocephalus based upon a survey of 284 cases with report of 11 additional cases. Acta Neurochir(Wien) 16:1–78, 1967.

Porter JM, Pidgeon C, Cunningham AJ. The sitting position in neurosurgery: a critical appraisal. Br J Anaesth 82:117–128, 1999.

Reasoner DK, Todd MM, Scamman FL, Warner DS. The incidence of pneumocephalus after supratentorial craniotomy. Observations on the disappearance of intracranial air. Anesthesiology 80:1008–1012, 1994.

Standefer M, Bay JW, Trusso R. The sitting position in neurosurgery: a retrospective analysis of 488 cases. Neurosurgery 14:649–658, 1984.

Stroop JR. Studies of interference in serial verbal reactions. J Exp Psycho 18: 643–662, 1935.

Toung T, Donham RT, Lehener A, Alano J, Campbell J. Tension pneumocephalus after posterior fossa craniotomy: report of four additional cases and review of postoperative pneumocephalus. Neurosurgery 12:164–168, 1983.

## Color-word interference test

(J. R. Stroop, 1935)

The Stroop color-word interference test is a useful and reliable assessment tool for the identification of executive function deficits. As the basic principle of Stroop test is competition of semantic meaning and font font color, subjects were shown colored words with conflicting visual and verbal information which led to interference in word and color meanings.

First task – word name

Study participants should read the written color names of the words independently of the color of the ink as fast as possible. The time will be measured by a stop-watch. Mistakes will be marked (X over the words).

Second task – color name

Study participants should name the color of the letters independently of the written word as fast as possible. The time will be measured by a stop-watch. Mistakes will be marked (X over the words).

## Color-word interference test (preoperative)

First task – word name

Please read the written color names of the words independently of the color of the ink as fast as possible. The time will be measured and mistakes will be marked (X over the words).

Name: _________________________ Date: __________ ___________

| **Red** | **Blue** | **Yellow** | **Green** | **Yellow** | **Green** | **Yellow** | **Red** | **Green** | **Red** |
| --- | --- | --- | --- | --- | --- | --- | --- | --- | --- |
| **Blue** | **Green** | **Red** | **Yellow** | **Red** | **Green** | **Green** | **Blue** | **Red** | **Yellow** |
| **Green** | **Blue** | **Red** | **Blue** | **Blue** | **Red** | **Green** | **Blue** | **Green** | **Blue** |
| **Yellow** | **Green** | **Red** | **Green** | **Yellow** | **Red** | **Blue** | **Red** | **Blue** | **Red** |
| **Red** | **Yellow** | **Red** | **Green** | **Green** | **Blue** | **Red** | **Yellow** | **Blue** | **Green** |

**Time: First task ____________ sec Mistakes: First task ___________**

**Investigator: _____________________**

## Color-word interference test (preoperative)

Second task – color name

Please name the color of the letters independently of the written word as fast as possible. The time will be measured and mistakes will be marked (X over the words).

Name: _________________________ Date: __________ ___________

| **Red** | **Blue** | **Yellow** | **Green** | **Yellow** | **Green** | **Yellow** | **Red** | **Green** | **Red** |
| --- | --- | --- | --- | --- | --- | --- | --- | --- | --- |
| **Blue** | **Green** | **Red** | **Yellow** | **Red** | **Green** | **Green** | **Blue** | **Red** | **Yellow** |
| **Green** | **Blue** | **Red** | **Blue** | **Blue** | **Red** | **Green** | **Blue** | **Green** | **Blue** |
| **Yellow** | **Green** | **Red** | **Green** | **Yellow** | **Red** | **Blue** | **Red** | **Blue** | **Red** |
| **Red** | **Yellow** | **Red** | **Green** | **Green** | **Blue** | **Red** | **Yellow** | **Blue** | **Green** |

**Time: Second task ____________ sec Mistakes: Second task ___________**

**Investigator: _____________________**

## Color-word interference test (postoperative)

First task – word name

Please read the written color names of the words independently of the color of the ink as fast as possible. The time will be measured and mistakes will be marked (X over the words).

Name: _________________________ Date: __________ ___________

| **Red** | **Blue** | **Yellow** | **Green** | **Yellow** | **Green** | **Yellow** | **Red** | **Green** | **Red** |
| --- | --- | --- | --- | --- | --- | --- | --- | --- | --- |
| **Blue** | **Green** | **Red** | **Yellow** | **Red** | **Green** | **Green** | **Blue** | **Red** | **Yellow** |
| **Green** | **Blue** | **Red** | **Blue** | **Blue** | **Red** | **Green** | **Blue** | **Green** | **Blue** |
| **Yellow** | **Green** | **Red** | **Green** | **Yellow** | **Red** | **Blue** | **Red** | **Blue** | **Red** |
| **Red** | **Yellow** | **Red** | **Green** | **Green** | **Blue** | **Red** | **Yellow** | **Blue** | **Green** |

**Time: First task ____________ sec Mistakes: First task ___________**

**Investigator: _____________________**

## Color-word interference test (postoperative)

Second task – color name

Please name the color of the letters independently of the written word as fast as possible. The time will be measured and mistakes will be marked (X over the words).

Name: _________________________ Date: __________ ___________

| **Red** | **Blue** | **Yellow** | **Green** | **Yellow** | **Green** | **Yellow** | **Red** | **Green** | **Red** |
| --- | --- | --- | --- | --- | --- | --- | --- | --- | --- |
| **Blue** | **Green** | **Red** | **Yellow** | **Red** | **Green** | **Green** | **Blue** | **Red** | **Yellow** |
| **Green** | **Blue** | **Red** | **Blue** | **Blue** | **Red** | **Green** | **Blue** | **Green** | **Blue** |
| **Yellow** | **Green** | **Red** | **Green** | **Yellow** | **Red** | **Blue** | **Red** | **Blue** | **Red** |
| **Red** | **Yellow** | **Red** | **Green** | **Green** | **Blue** | **Red** | **Yellow** | **Blue** | **Green** |

**Time: Second task ____________ sec Mistakes: Second task ___________**

**Investigator: _____________________**

**Stanford Sleepiness Scale**

( Hoddes et al., 1973 )

Please decide using the point-scale below what best represents how you are feeling and mark the corresponding number on the chart below.

Name: _________________________ Date: __________ ___________

| **Degree of sleepiness** | **Scale**  **Rating** |
| --- | --- |
| Feeling active, vital, alert, or wide awake | 1 |
| Functioning at high levels, but not fully alert | 2 |
| Awake, but relaxed; responsive but not fully alert | 3 |
| Somewhat foggy, let down | 4 |
| Foggy; losing interest in remaining awake; slowed down | 5 |
| Sleepy, woozy, fighting sleep; prefer to lie down | 6 |
| No longer fighting sleep, sleep onset soon; having dream-like thoughts | 7 |
| Asleep | X |

**Investigator : ______________________**

**STUDY FLOW CHARTS**

**Study title:** Normobaric hyperoxia for treatment of pneumocephalus after posterior fossa surgery in the

semisitting position: a prospective randomized controlled trial

**Patient’s selection, Informed consent, Stroop test**

**Surgery, Wound closure**

**ABG**

**< 2 hours**

**Cranial CT scan with image reconstruction**

**Intracranial air collection of 30 ml or more?**

**Yes No excluded**

**randomization**

**Group 1 Group 2**

**Ventilated with FiO_2_ 100’% weaned off anaesthesia,**

**via the ETT extubation, ABG**

**3 hours**

**weaned off anaesthesia,**

**extubation, ABG**

**If necessary, further O_2_**

**via the ETT**

**primary endpoints:**

**cranial CT scan at 24 h after ventilation with FiO_2_ 100’%**

**secondary endpoints:**

**Stanford Sleepiness Scale,**

**Stroop test**

**PATIENT INFORMATION SHEET**

**Study title:** Normobaric hyperoxia for treatment of pneumocephalus after posterior fossa surgery in the

semisitting position: a prospective randomized controlled trial

Dear Madam,

Dear Sir,

at Hannover Medical School, we aim to constantly improve patient care. For this purpose, clinical studies are important and necessary. We currently perform a clinical study on the effect of normobaric hyperoxia for treatment of pneumocephalus after posterior fossa surgery in the semisitting position.

You have been chosen as a candidate for this clinical study since you are scheduled to undergo surgery in semisitting position due to your current medical condition. The semisitting position has been used widely for posterior fossa surgery. There are, however, some potential complications associated with surgery in the semisitting position such as intracranial air accumulations, so-called pneumocephalus. Small amounts of intracranial air are reabsorbed completely within days or weeks, and in most instances, no clinical signs or symptoms are noted. Accumulation of large amounts of intracranial air can occupy intracranial space, which may result in various symptoms, e.g. headache, or in extreme cases, deterioration of consciousness with potentially life-threatening brainstem compression. Previous studies indicated a possible beneficial effect of administration of normobaric oxygen at FiO_2_ 100% on the absorption of postoperative pneumocephalus. We aim to prove the efficacy of normobaric hyperoxia at FiO_2_ 100% on resorption of postoperative pneumocephalus. Since there is no study yielding higher class evidence on the application of oxygen for treatment of pneumocephalus, we decided to perform a prospective randomized study. The result we get from this study will help us treat future patients with postoperative pneumocephalus with the most appropriate treatment.

If you enter the study you will first of all be asked to perform Stroop color-word interference test prior to surgery. A postoperative cranial CT scan will be performed immediately after completion of surgery. You will be finally included in the study and the randomization process if a routine postoperative CT scan would show a volume of intracranial air of 30 ml or more. If you are allocated to the treatment group, you will be ventilated with FiO_2_ = 100% via the endotracheal tube for a period of 3 hours, starting immediately after admission to the neurosurgical intensive care unit.

A second CT scan and Stroop color-word interference test will be performed about 24 hours after surgery. Furthermore, you will be asked to indicate your level of sleepiness according to the Stanford Sleepiness Scale. No adverse effects are expected after administration of normobaric hyperoxia in time duration of 3 hours.

###### Protection of data privacy

All medical records, data, and the results collected during the study will be processed as follows:

The records that identify you will be collected by a study doctor. The informed consent will be kept confidential. All medical records, data, and the results collected during the study will be labeled with your anonymous subject number and will be stored on a computer. Only your study doctor will know the information is related to you. The results of the study will be published in medical literature and/or presented at a scientific conference or symposium, but your identity will not be revealed.

The study related data will be stored until the results are published. Thereafter the data will be deleted. The study related medical records will be kept for 30 years and thereafter will be deleted.

If you have any questions as a participant in this study, you can contact one of the following Investigators:

PD Dr. med. M. Nakamura, Department of Neurosurgery, Tel. 0511–532 3770.

Dr. med. E. J. Hermann, Department of Neurosurgery, Tel. 0511–532 2359.

B. Hong, Department of Neurosurgery, Tel. 0511–532 3101.

Taking part in this study is voluntary. If you decide to take part in the study, you are free to withdraw from the study at any time without giving any reason and without your medical care or legal rights being affected. If you do not want to take part in this study or if you wish to withdraw from the study at any time you may do so without giving a reason and you will not lose any benefits to which you would otherwise be entitled. If you withdraw from the study the data collected will not be analyzed for the purpose of the study.

**INFORMED CONSENT FORM**

**Study title:** Normobaric hyperoxia for treatment of pneumocephalus after posterior fossa surgery in the

semisitting position: a prospective randomized controlled trial

I have been informed about this clinical study by Dr. ...........................

I confirm that I have had time to carefully read and understand the patient information sheet provided for this study. I confirm that I have had the opportunity to discuss the study and ask questions and I am satisfied with the answers and explanations that I have been provided.

I understand that my participation is voluntary and that I am free to withdraw at any time without giving any reason and without my medical care or legal rights being affected. I give permission for my medical records to be reviewed by the Investigators. The records that identify a patient will be kept confidential and regarding to applicable laws and regulations, will not be made publicly available.

### Hannover, date

______________________________________ ______________________________________

( Investigator ) ( Patient )
